# Intragenic repeat expansion in the cell wall protein gene *HPF1* controls yeast chronological aging

**DOI:** 10.1101/gr.253351.119

**Published:** 2020-05

**Authors:** Benjamin P. Barré, Johan Hallin, Jia-Xing Yue, Karl Persson, Ekaterina Mikhalev, Agurtzane Irizar, Sylvester Holt, Dawn Thompson, Mikael Molin, Jonas Warringer, Gianni Liti

**Affiliations:** 1 Université Côte d'Azur, CNRS, INSERM, IRCAN, 06107 Nice, France;; 2 Department of Chemistry and Molecular Biology, University of Gothenburg, 41390 Gothenburg, Sweden;; 3 Ginkgo Bioworks Incorporated, Boston, Massachusetts 02210, USA;; 4 Department of Biology and Biological Engineering, Chalmers University of Technology, 41296 Gothenburg, Sweden

## Abstract

Aging varies among individuals due to both genetics and environment, but the underlying molecular mechanisms remain largely unknown. Using a highly recombined *Saccharomyces cerevisiae* population, we found 30 distinct quantitative trait loci (QTLs) that control chronological life span (CLS) in calorie-rich and calorie-restricted environments and under rapamycin exposure. Calorie restriction and rapamycin extended life span in virtually all genotypes but through different genetic variants. We tracked the two major QTLs to the cell wall glycoprotein genes *FLO11* and *HPF1*. We found that massive expansion of intragenic tandem repeats within the N-terminal domain of *HPF1* was sufficient to cause pronounced life span shortening. Life span impairment by *HPF1* was buffered by rapamycin but not by calorie restriction. The *HPF1* repeat expansion shifted yeast cells from a sedentary to a buoyant state, thereby increasing their exposure to surrounding oxygen. The higher oxygenation altered methionine, lipid, and purine metabolism, and inhibited quiescence, which explains the life span shortening. We conclude that fast-evolving intragenic repeat expansions can fundamentally change the relationship between cells and their environment with profound effects on cellular lifestyle and longevity.

Aging is a progressive decline in biological functions occurring in almost all living organisms that ultimately leads to death (Finch 1990; Jones et al. 2014). The first life span regulating genes were identified in the beginning of the 1990s (Johnson 1990; Kenyon et al. 1993; Sun et al. 1994). Today, hundreds have been uncovered (Kenyon 2010), although most are of small effect and few explain aging variation between individuals. Besides genetics, environmental factors, such as calorie restriction (CR) (Klass 1977; Weindruch et al. 1986; Jiang 2000; Pletcher et al. 2002; Colman et al. 2009), reduced oxygen exposure (Rascon and Harrison 2010; Leiser et al. 2013), and low temperature (Sestini et al. 1991; Conti et al. 2006; Leiser et al. 2011), extend longevity. How genetics and environment interact to control variation in life span and by which mechanisms remains poorly understood. The beneficial effect of calorie restriction on longevity in organisms ranging from yeast (Lin et al. 2000) to primates (Mattison et al. 2017) has been known for >80 yr (McCay et al. 1935) and is still the most successful intervention to delay aging, although its impact on life span has sometimes been disputed (Liao et al. 2010; Schleit et al. 2013). Cellular mediation of CR is at least in part occurring through nutrient-sensitive signaling networks, including the insulin/IGF1, MTOR (target of rapamycin), cAMP-PKA, and AMPK pathways. These regulate life span by controlling stress responses, mitochondrial respiration, redox homeostasis, genome stability, autophagy, energy, and fat metabolism (Schulz et al. 2007; Weinberger et al. 2007; Hansen et al. 2008; Madia et al. 2008; Wei et al. 2008; Alvers et al. 2009; Molin et al. 2011; Yuan et al. 2012). Pharmaceutical control of some of these pathways can extend longevity in model organisms. Rapamycin, a clinically approved TOR inhibitor (Eisenberg et al. 2009; Harrison et al. 2009; Martin-Montalvo et al. 2013; De Haes et al. 2014), extends life span by mimicking CR (Blagosklonny 2010), but undesirable side effects in humans restrict its usage (Kaeberlein 2014).

The budding yeast *Saccharomyces cerevisiae* has been pivotal in elucidating mechanisms regulating aging. Yeast aging can be studied through two approaches: replicative life span (RLS) and chronological life span (CLS). Replicative lifespan is the number of mitotic divisions before senescence and is used as a paradigm to study aging of proliferative tissues, such as stem cells (Mortimer and Johnston 1959; Steinkraus et al. 2008). Chronological life span is the time yeasts survive in nonproliferative conditions and models the aging of post-mitotic cells, such as neurons (Longo et al. 1996, 2012). Hundreds of genes whose disruption affects the CLS of lab-domesticated yeast in calorie-rich (Powers et al. 2006; Fabrizio et al. 2010; Garay et al. 2014) and calorie-restricted (Matecic et al. 2010; Campos et al. 2018) environments have been identified. However, most studies relied on artificial gene deletions and were performed in lab-domesticated strains, which are highly atypical (Warringer et al. 2011), maintained as haploids rather than diploids (Peter et al. 2018), carry auxotrophies that alter life span (Gomes et al. 2007; Boer et al. 2008), and have never been exposed to natural selection (Kaya et al. 2015). Thus, genetic variants that control natural life span variation are still largely unknown. Crosses between natural yeast strains have the potential to uncover these variants (Brem 2002; Steinmetz et al. 2002) but remain poorly explored. Previous work linked natural polymorphisms in the ribosomal DNA and in the sirtuin *SIR2* (Stumpferl et al. 2012; Kwan et al. 2013), as well as telomere maintenance (Kwan et al. 2011) and serine biosynthesis (Jung et al. 2018) to life span variation. Lack of genetic diversity, mapping resolution, and power has prevented more exhaustive exploration. Here, we leveraged a highly recombined *S. cerevisiae* population derived from natural isolates with the aim to map genetic variants controlling yeast chronological life span. We generated a thousand unique diploid individuals and monitored their longevity with and without treatments promoting life span to provide a detailed portrait of naturally occurring yeast life span variants.

## Results

### Calorie restriction and rapamycin extend life span through different genetic variants

We crossed a long-lived North American (NA) oak tree bark strain (YPS128) with a short-lived West African (WA) palm wine strain (DBVPG6044) which differ at 0.53% of nucleotide sites (Liti et al. 2009; Parts et al. 2011). A pool of F12 segregants of opposite mating types were then mated to generate 1056 diploids with hybrid, phased genomes, termed Phased Outbred Lines (POLs) (Hallin et al. 2016). POLs were individually cultivated in calorie-rich (synthetic dextrose complete, SDC), calorie restricted, or rapamycin-supplemented (RM) environments for the whole experiment, and viability was measured by high throughput flow cytometry at 7, 21, and 35 d after media exhaustion. A total of 52,466 genetic markers were called and used to run a genome-wide linkage analysis.

**Figure 1.**
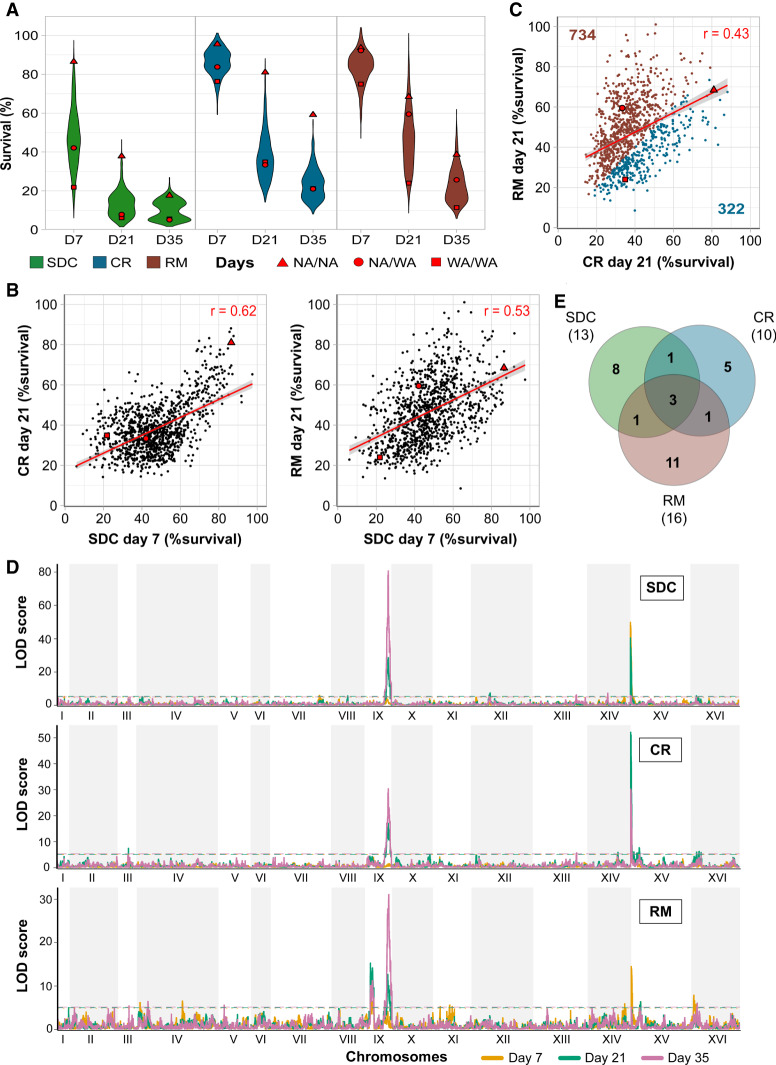
Calorie restriction and rapamycin extend life span through different genetic variants. Chronological life span of 1056 diploid segregant lineages from an F12 NA/WA advanced intercross. CLS was measured by counting viable cells (%) 7, 21, and 35 d after entry into quiescence, following growth in calorie-sufficient (SDC), -restricted (CR), and rapamycin (RM) media. Red: Founder homozygote parents (NA/NA, WA/WA) and their F1 hybrid (NA/WA). (*A*) CLS distributions across time points and conditions. (*B*) Comparing CLS across environments and time points. Red line: linear regression, with 95% confidence interval. (*C*) CLS comparison in RM and CR. Numbers: lineages living longer in CR (blue) or RM (brown). (*D*) Linkage analysis of CLS. Panels: calorie-rich (*top*), -restricted (*middle*), and rapamycin (*bottom*) media. Line color: 7 (yellow), 21 (green), and 35 (purple) days after entry into quiescence. *y*-axis: LOD score, *x*-axis: genome position. Dashed lines: Significance QTL (α = 0.05). (*E*) QTLs private to and shared between environments. Numbers in parentheses: total QTLs per environment.

We found a broad lifespan diversity (Fig. 1A; Supplemental Table S3), with survival rates ranging from 6% to 97% already after 7 d in SDC (47% mean viability). Calorie restriction (86% mean viability at day 7) and RM (83% mean viability at day 7) extended life spans of all genotypes (with a single exception in RM) (Fig. 1A; Supplemental Fig. S1A). Life span was fairly correlated across environments (Pearson's *r* = 0.62 for SDC vs. CR, 0.53 for SDC vs. RM) (Fig. 1B), implying that CLS is mainly regulated by shared genetic effects across environments. Nevertheless, the more modest correlation between CR and RM (Pearson's *r* = 0.43) (Fig. 1C) suggested that they extend life span through partially distinct mechanisms.

We found a total of 30 unique QTLs associated with chronological aging (Fig. 1D; Supplemental Fig. S1B) that explained up to 40% of life span variation. QTLs were mostly private to one environment, and only three were detected in all (Fig. 1E). Two of these, located on Chr IX and Chr XV, were stronger than others and explained up to ∼30% and ∼20% of life span variation, respectively (Supplemental Table S4). Although both major QTLs were ubiquitous, the Chr XV QTL was partially masked by RM treatment, whereas the Chr IX QTL became significant only at advanced age (days 21 and 35). Most of the remaining QTLs were time- and environment-dependent (Fig. 1E; Supplemental Fig. S1C) and explained much less (mean: ∼3%) of the life span variation (Supplemental Table S4). Thus, CLS was largely determined by a few, very strong QTLs that were shared across calorie-rich and calorie-restricted environments. Chronological life span was then fine-tuned by mechanisms private to each environment, although the conservative threshold for calling QTLs may lead us to somewhat underestimate the shared QTLs.

### Natural variations in the cell wall glycoproteins Hpf1p and Flo11p control chronological life span

**Figure 2.**
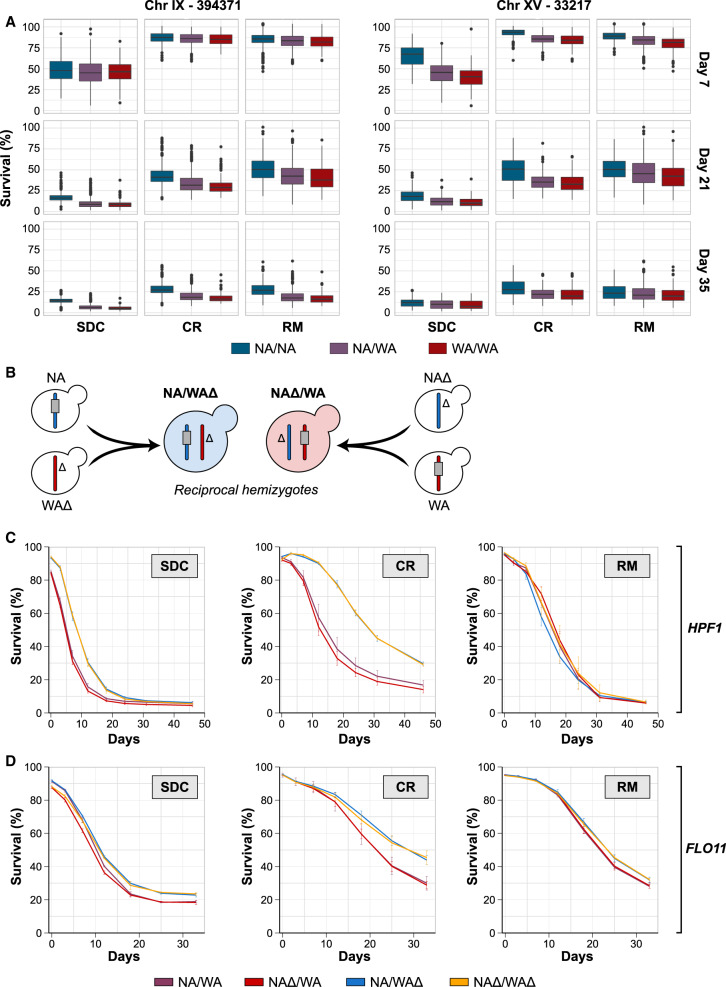
Natural allelic variations in the *HPF1* and *FLO11* control chronological life span. (*A*) Chronological life span of the 1056 POLs separated according to genotype at the markers with highest LOD score in each of the two major QTLs: 394,381 kb in Chromosome IX (in *FLO11*) and 33,217 kb in Chromosome XV (in *HPF1*). (*B*) Schematic representation of the NA/WA reciprocal hemizygosity design used to validate the CLS effect of the *HPF1* and *FLO11* WA alleles. Color: NA (blue) and WA (red) chromosomes. Gray rectangle: candidate gene (*HPF1, FLO11*), Δ: gene deletion. (*C*) Reciprocal hemizygosity. CLS of NA/WA hemizygotes for NA (blue; WAΔ) and WA (red; NAΔ) *HPF1*, heterozygote for *HPF1* (purple; NA/WA), and lacking *HPF1* (yellow; NAΔ/WAΔ). (*D*) As in *C*, but for *FLO11*.

The two major QTLs peaked within *FLO11* (Chr IX) and *HPF1* (Chr XV). Both encode secreted cell wall glycoproteins with no known connections to life span. Hpf1p is functionally uncharacterized, while Flo11p regulates cell adhesion, pseudohyphae, and biofilm formation (Guo et al. 2000; Douglas et al. 2007; Váchová et al. 2011). We found a shorter life span for WA-*FLO11* and *HPF1* compared to NA homozygotes, with the WA short life span alleles being completely dominant (Fig. 2A). We validated these effects in a reciprocal hemizygosity assay (Fig. 2B; Methods; Steinmetz et al. 2002); a NA/WA hybrid deleted for the WA-*HPF1* allele lived 70% longer, regardless of the presence of NA-*HPF1* (Fig. 2C). Life span extension by rapamycin, but not by calorie restriction, rescued the WA-*HPF1* induced life span shortening, consistent with the QTL being weaker in rapamycin (Fig. 2C,D). The WA-*FLO11* also shortened life span but less than expected from the QTL strength, likely due to linkage or epistasis with other variants. As for *HPF1*, removing the WA-*FLO11* or both the WA and NA-*FLO11* alleles extended life span, while removing the NA-*FLO11* had no effect (Fig. 2D). The negative effect of WA-*FLO11* increased with age and was not rescued by rapamycin, again as expected from linkage analysis (Figs. 2D, 1D). Removing both alleles of either *HPF1* or *FLO11* extended the life span of WA/WA but not NA/NA homozygotes and had no effect in the domesticated reference strain, S288C (Supplemental Fig. S2A–C). We found one additional major RM-specific QTL located on the left arm of Chr IX. We tested four candidate genes (*FKH1*, *ASG1*, *RPL16A*, *RPI1*) located close to the QTL peak by reciprocal hemizygosity; however, allelic variation in these genes had no effect on CLS (Supplemental Fig. S3). The width of this QTL (40 kb, >30 genes) makes identifying the causative variants challenging.

### Massive intragenic tandem repeat expansions within *HPF1* shorten life span

**Figure 3.**
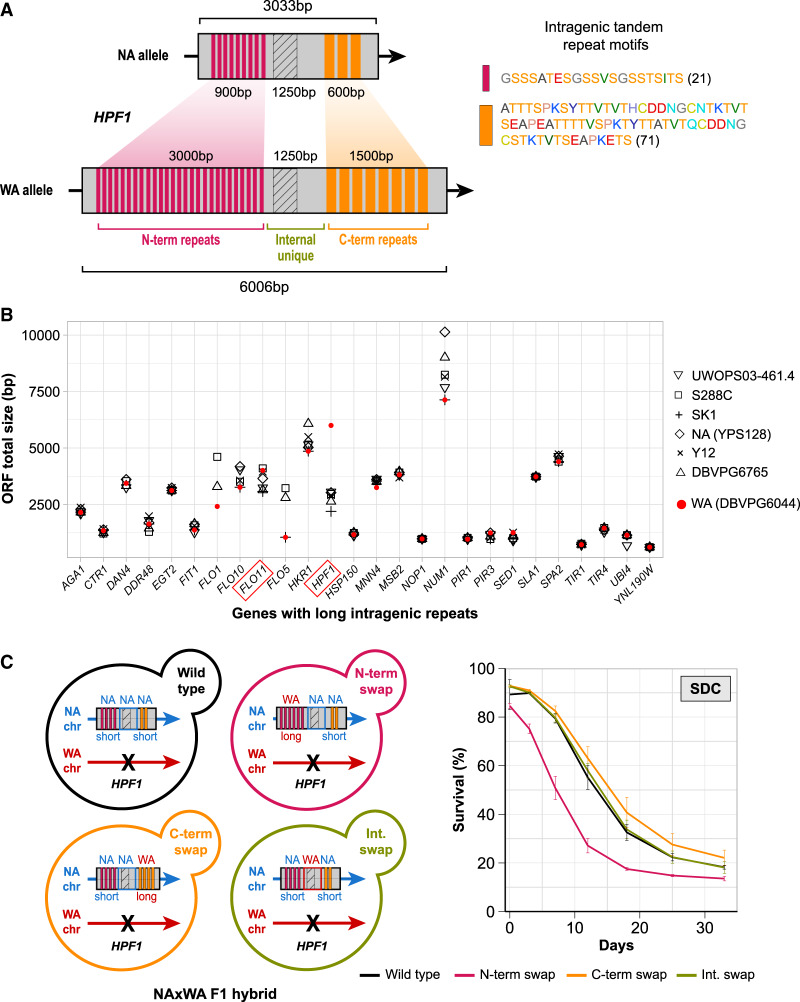
Massive intragenic tandem repeat expansions within *HPF1* shorten life span. (*A*) Schematic representation of the intragenic repeats (colored rectangles) in *HPF1* for NA and WA alleles. Hatched rectangle: a *HPF1* internal unique region with high sequence variation between NA and WA alleles. *Right*: repeat motif units. Amino acids are colored according to the RasMol nomenclature. Numbers = motif size (amino acids). (*B*) Size variation of genes containing long intragenic repeats in seven diverged *S. cerevisiae* strains (Verstrepen et al. 2005; Yue et al. 2017). Diamonds: North American, red circles: West African. (*C*) *Left* panel: Design of allele swaps of *HPF1* segments in the NA/WA F1 hybrid. The WA-*HPF1* allele was deleted (black cross), while the NA-*HPF1* was kept unchanged (wild type), or a segment was replaced by the corresponding WA-*HPF1* segment. N-term: N-terminal repeats, C-term: C-terminal repeats, Int: internal unique region. *Right* panel: CLS for allele swapped constructs in SDC media.

Inspecting complete genome assemblies, we found that *FLO11* and *HPF1* both carry intragenic tandem repeats that are expanded in the WA allele. WA-*HPF1* is twice as long (6006 vs. 3033 bp) and WA-*FLO11* is 10% longer (4014 vs. 3654 bp) than their NA counterparts (Fig. 3A; Supplemental Fig. S4A,B; Supplemental Table S6). Repeat motifs were between 21 and 71 amino acids and mainly composed of threonine and serine, as reported for repeat motifs in other cell wall proteins (Fig. 3A; Verstrepen and Klis 2006). The partial degeneration of WA-*HPF1* and WA-*FLO11* repeat motifs prevented pinpointing their exact patterns and boundaries. Such large repeat expansions in the WA isolate is specific to *HPF1*; of 26 genes containing very long tandem repeats (Verstrepen et al. 2005), only *HPF1* was massively expanded in WA relative to six other strains for which complete genome assemblies exist (Fig. 3B; Yue et al. 2017).

We hypothesized that the WA-*HPF1* massive repeat expansions explained the life span shortening and tested this by swapping *HPF1* alleles in the F1 NA/WA hybrid. We removed WA-*HPF1*, while we engineered the remaining NA allele to contain specific segments of the WA allele (see Methods). Swapped segments corresponded to N- and C-terminal blocks of tandem repeats and to the highly polymorphic internal unique domain (Fig. 3A,C). Substituting the C-terminal repeats or the internal domain of NA-*HPF1* with its WA counterpart did not shorten CLS, but replacing the N-terminal repeats shortened CLS as much as the native WA allele (Fig. 3C). Likewise, inserting the WA N-terminal repeats into a NA homozygote diploid shortened life span, while inserting the NA N-terminal repeats into a WA homozygote diploid extended its life span to a comparable extent (Supplemental Fig. S4C). We performed the same assay for *FLO11*, shifting the complete array of WA-*FLO11* tandem repeats into the NA allele in the NA/WA hybrid background while removing WA-*HPF1*. In contrast to what we observed in *HPF1*, insertion of WA-*FLO11* repeats had no effect on CLS, meaning that the life span shortening caused by WA-*FLO11* is driven by variants other than its repeat expansions (Supplemental Fig. S4D).

### Buoyancy triggered by *HPF1* N-terminal repeat expansion impairs quiescence

**Figure 4.**
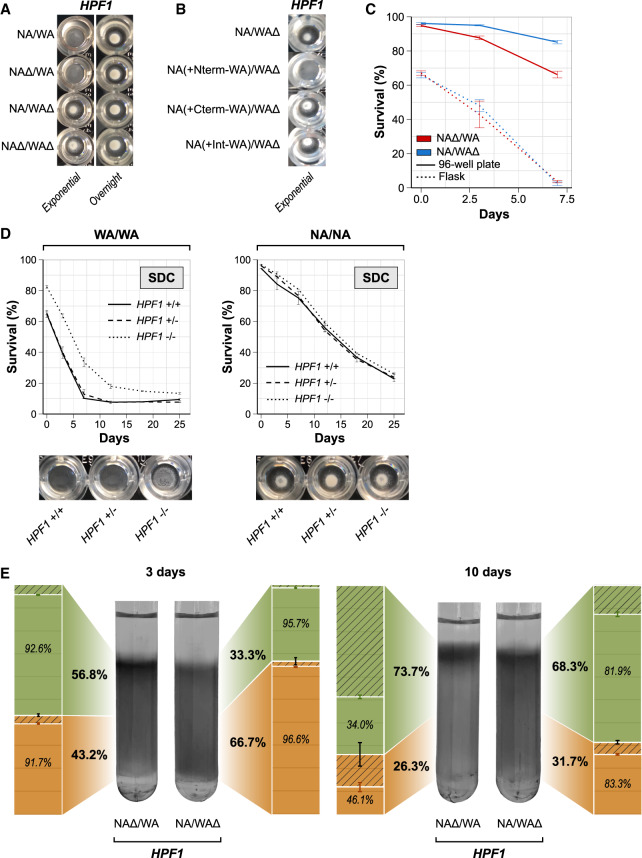
Buoyancy triggered by *HPF1* N-terminal repeat expansions shortens life span. (*A*,*B*) Buoyancy of cells cultivated for 7 h (exponential phase) or overnight in calorie-rich medium in a 96-well plate. (*A*) *HPF1* hemizygotes. (*B*) *HPF1* allele swaps (as described in Fig. 3C). (*C*) Comparing CLS of *HPF1* hemizygote cells cultivated in shake flasks and 96-well plates. Shake flasks had a 1:5 medium/volume ratio and were shaken at 220 rpm. Ninety-six-well plates were filled with 200 µL medium, with no shaking. (*D*) CLS and buoyancy (96-well plates; exponential phase) of WA and NA homozygotes parents with no (full line), 1 (dashed lines), or both copies (dotted lines) of *HPF1* deleted in calorie-rich medium. (*E*) Percoll density gradients with *HPF1* hemizygotes incubated in SDC media in a 96-well plate for either 3 d (*left* panel) or 10 d (*right* panel). The upper (nonquiescent cells) and lower (quiescent cells) phases were isolated by pipetting. The fraction (bold) and viability (italics) of cells in each phase were measured by flow cytometry (bar plots). Green: Upper/nonquiescent fraction, orange: lower/quiescent fraction, hatched area: dead cell fraction.

We observed that NA/WA hybrids carrying the WA-*HPF1* became buoyant during exponential growth*,* i.e., they shifted to a free-floating lifestyle. Following entry into stationary phase, cells sedimented again, returning to a sedentary lifestyle (Fig. 4A). *FLO11* alleles had no effect on buoyancy (Supplemental Fig. S5A). We showed that the shift to a buoyant lifestyle was caused by the WA-*HPF1* N-terminal repeat expansion (Fig. 4B). To probe whether buoyancy per se shortens lifespan, we repeated the CLS assay under conditions of high aeration in intensely shaken flasks rather than in static 96-well plates. Because intense shaking forces all yeast cells to remain in suspension, we postulated that it would eliminate the *HPF1* allelic effect only if it was due to buoyancy. In line with this assumption, we found that buoyancy enforced by shaking reduced life span and completely negated the effect of *HPF1* allelic variation on life span (Fig. 4C). A shorter life span in shaking cultures has previously been explained as a result of higher exposure to oxygen (Longo et al. 1999; Fabrizio et al. 2003), although a too low access to oxygen also impairs the life span by reducing mitochondrial respiration (Ocampo et al. 2012). We therefore probed whether increased oxygen exposure as a consequence of a buoyant lifestyle could explain the shorter life span of WA-*HPF1* cells. We cultivated cells with WA and NA *HPF1* alleles, respectively, in static, sealed tubes and varied the volume of air in each tube. Cultivation with lower air volume, and therefore with lesser oxygen exposure, completely suppressed the shorter life span of cells with the WA-*HPF1* allele (Supplemental Fig. S5B). Thus, by promoting cellular buoyancy in static cultures, WA-*HPF1* exposes cells to higher oxygen levels and shortens life span. Neither secretion of Hpf1p to the medium nor variations in medium acidity had any life span effect (Supplemental Fig. S5C,D). Complete *HPF1* removal in the parental WA homozygote nullified the shift to a buoyant lifestyle and increased CLS, while *HPF1* removal affected neither buoyancy nor life span in the parental NA homozygote background (Fig. 4D).

Higher external oxygen increases intracellular superoxide anion levels and thereby prevents cells from arresting growth and entering into the aging-resistant quiescent state upon nutrient exhaustion (Weinberger et al. 2010). We therefore tested whether WA-*HPF1*-driven buoyancy impairs quiescence. We measured both the fraction and viability of quiescent cells in *HPF1* reciprocal hemizygotes, using density gradients (Allen et al. 2006). We found a higher fraction of quiescent cells in the sedentary NA-*HPF1* hemizygote than in the buoyant WA-*HPF1* 3 d post-inoculation (Fig. 4E). After 10 d, the number of quiescent cells decreased for both hemizygotes, as previously observed (Madia et al. 2009). At this stage, we found only a minor viability difference between upper and lower phases (Fig. 4E), possibly because the enrichment for quiescent cells in the lower phase is less pronounced in natural than in lab strains or because there may be multiple quiescence states with distinct cell properties (Klosinska et al. 2011; Miles et al. 2019).

Many cell wall proteins are involved in cell-cell cohesion, cell-surface adhesion, often through pseudohyphal growth, which could affect buoyancy (Lo and Dranginis 1998; Verstrepen and Klis 2006). However, we found no role of *HPF1* in flocculation or pseudohyphal growth (Supplemental Figs. S5E, S6). Complete loss of *HPF1* decreased surface adhesion in the NA homozygote parent (Supplemental Fig. S7). Because neither the WA homozygote nor the NA/WA hybrid adhered to surfaces, we could not test if *HPF1* reduced surface adhesion in these backgrounds (Supplemental Fig. S7). Overall, the lack of these traits in the NA/WA hybrid background rules out their contribution to buoyancy and life span.

### *HPF1*-induced buoyancy reprograms methionine, lipid, and purine metabolism

**Figure 5.**
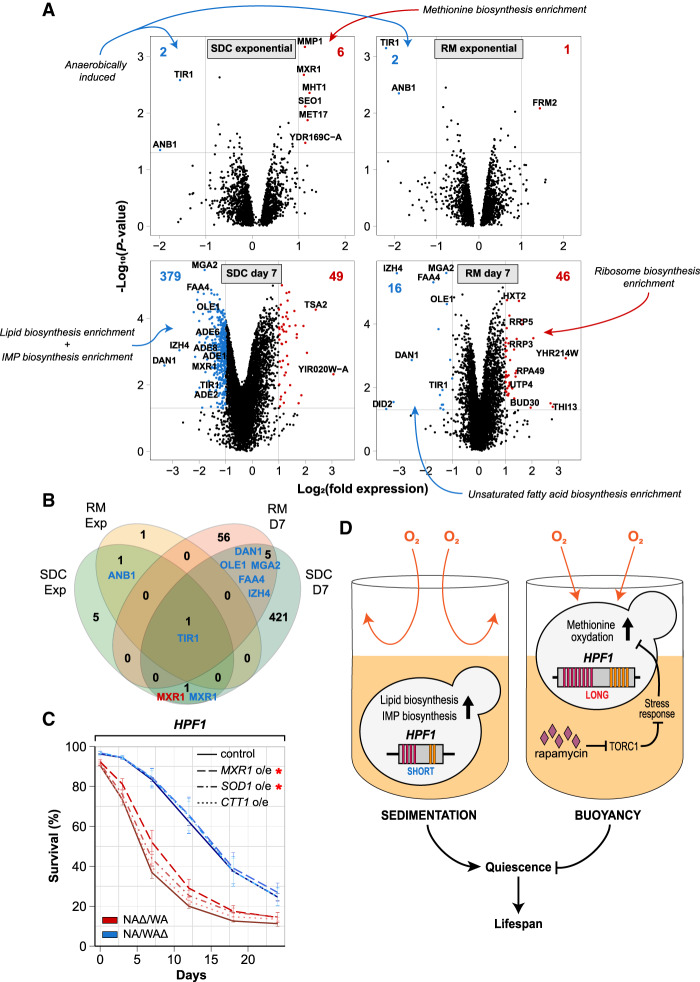
*HPF1*-induced buoyancy reprograms methionine, lipid, and purine metabolism. (*A*) Transcriptome changes induced by WA-*HPF1*-dependent buoyancy. NA/WA hybrids hemizygotes for WA or NA-*HPF1* were cultivated in calorie-rich (*left* panels) or rapamycin (*right* panels) medium, and RNA was extracted and sequenced from exponential phase (*top* panels) or aging (*bottom* panels; 7 d after entry into quiescence) cells. *y*-axis: −log_10_ (*P*-value), *x*-axis: log_2_ (*HPF1* NAΔ/WA) – log_2_ (*HPF1* NA/WAΔ). Blue: transcripts more (>2×, *P* < 0.05) abundant in *HPF1* NA/WAΔ, red: transcripts more (>2×, *P* < 0.05) abundant in *HPF1* NAΔ/WA. The total number of transcripts passing each criterion are reported (*top* corners, blue and red text). Gene Ontology classifications enriched among transcripts passing each criterion are indicated (blue and red arrows) (Supplemental Table S5). (*B*) Comparing the number of transcripts more (>2×, *P* < 0.05) abundant in *HPF1* NA/WAΔ (blue) or in *HPF1* NAΔ/WA (red) across environments (SDC and RM, in exponential phase and after 7 d of aging [D7]). (*C*) CLS of *HPF1* reciprocal hemizygotes overexpressing (o/e) either *MXR1* (dashed), *SOD1* (dot-dashed), or *CTT1* (dotted) in SDC media in 96-well plate. Red asterisk indicates significance (*P* < 0.05 unpaired Student's *t*-test) at 7 d (*MXR1*) and 12 d (*MXR1* and *SOD1*). (*D*) Model for how the intragenic tandem repeat expansions in WA-*HPF1* shortens the life span by shifting cells from a sedentary (*left*) to a buoyant lifestyle (*right*), exposing them to more oxygen and causing mild oxidation of, in particular, methionine, which impairs entry into quiescence. Rapamycin prevents the accumulation of oxidized methionine, possibly through inhibiting TORC1 and through the activation of stress response genes.

We hypothesized that buoyant WA-*HPF1* cells exposed to higher oxygen experience altered cellular redox homeostasis and that this required reprogramming redox metabolism and gene expression. We therefore compared the transcriptomes of the two *HPF1* reciprocal hemizygous hybrids before (exponential growth) and after (7 d) the onset of aging, with or without rapamycin exposure (Supplemental Table S5). During exponential growth, buoyancy and higher oxygen exposure induced by the WA-*HPF1* only weakly affected relative transcript abundances (eight and three genes changing greater than twofold in calorie-rich and rapamycin media, respectively) (Fig. 5A,B). The known low oxygen responders *TIR1* and *ANB1* (Lowry and Lieber 1986; Cohen et al. 2001) were repressed by oxygen (i.e., in WA-*HPF1*). Five of the six transcripts induced by oxygenation encode proteins with a role in methionine metabolism (Fig. 5A), notably *MXR1*, which encodes a methionine-S-sulfoxide reductase known to control life span (Koc et al. 2004). In contrast, during chronological aging, the WA-*HPF1* cells expressed less *MXR1* (Fig. 5A,B). We overexpressed (over 10-fold on a transcript level) *MXR1* and found this overexpression to partially rescue the short life span of WA-*HPF1* cells (Fig. 5C). This suggests that the lower *MXR1* expression in WA-*HPF1* cells during chronological aging limited their life span. Overexpression of *SOD1,* encoding the mostly cytosolic superoxide dismutase, also improved the CLS of WA-*HPF1* cells somewhat, while overexpression of the cytosolic catalase *CTT1* did not (Fig. 5C). This further supports that the buoyant WA-*HPF1* cells experience an increased oxidative burden, although this is not enough to cause oxidative distress and reduce growth (Supplemantal Fig. S8A). We found that methionine restriction extended and methionine supplementation shortened the life span of cells (Supplemental Fig. S8B), as shown before (Johnson and Johnson 2014; Ruckenstuhl et al. 2014). Neither of these regimens suppressed the effect of WA-*HPF1*-induced buoyancy, however, suggesting that their premature aging was neither due to excessive nor deficient intracellular methionine pools. Rapamycin supplementation nullified the induction of methionine metabolism and partly restored *MXR1* expression, despite cells being buoyant, potentially explaining why rapamycin prevents the WA-*HPF1* from shortening life span (Figs. 5A,B,D, 2C).

In sharp contrast to before the onset of aging, we found the transcriptome to be fundamentally reprogrammed in WA-*HPF1* cells during aging (428 and 62 genes changing greater than twofold, in calorie-rich and rapamycin media) (Fig. 5A,B). The high oxygen exposure broadly repressed lipid and purine biosynthesis transcripts (Fig. 5A,D), whose expression is known to promote a long life span (Matecic et al. 2010; Garay et al. 2014; Handee et al. 2016; Arlia-Ciommo et al. 2018). *OLE1*, a known hypoxia responder encoding the fatty acid desaturase (Kwast et al. 1999), was much less expressed in the buoyant WA-*HPF1* hemizygote. Adding one of its main products, oleic acid, improved the CLS of both hemizygotes but could not rescue the short life span of WA-*HPF1*-induced buoyancy (Supplemental Fig. S8C).

## Discussion

We found calorie restriction to delay chronological aging for each of the 1056 yeast genotypes studied. This effect of calorie restriction is fully in line with reports on single gene knockouts, where calorie restriction extends chronological life span regardless of what gene is missing (Matecic et al. 2010). In contrast, calorie restriction only extends the replicative life span of approximately half of single gene knockouts, with many being negatively affected (Schleit et al. 2013). The diverging effects of calorie restriction on chronological and replicative life span may reflect the natural yeast life cycle; wild yeasts spend a significant part of their life span as starved, quiescent cells (Liti 2015; De Chiara et al. 2020) and survival in this state may be very strongly selected. In contrast, the mother-aged, extensively replicated state is extremely rare in natural yeast populations and unlikely to be under selection.

Except for the universally positive effects of calorie restriction, we found chronological life span to be extremely genotype-dependent, with a total of 30 distinct QTLs explaining up to 41% of life span variation. Among those, two major QTLs drove most of the life span variation (∼30% and ∼20%), while the remaining QTLs were more time- and environment-specific and contributed less (∼3% each). We linked the two major QTLs to the cell wall encoding genes *FLO11* (Chr IX) and *HPF1* (Chr XV), with West African alleles having a pronounced and dominant life span shortening effect. The cell wall integrity pathway reacts to cell wall perturbations, induced by, e.g., heat shock or starvation (Krause and Gray 2002), and regulates both chronological and replicative life spans (Kaeberlein and Guarente 2002; Ray et al. 2003; Stewart et al. 2007; Matecic et al. 2010). We found that the life span shortening induced by the WA-*HPF1* was not due to cell wall damage but to a shift in lifestyle. Cells carrying a North American *HPF1* had long, sedentary lives in sedimented yeast populations, while cells carrying a WA-*HPF1* lived shorter lives as buoyant, free-floating yeasts. The buoyancy directly caused the shorter life span, since forcing cells carrying the NA-*HPF1* to a free-floating state through vigorous shaking shortened the life span to the same levels as that of cells carrying the WA-*HPF1*. In contrast, we observed no buoyancy effect of *FLO11* variation, although *FLO11*-induced buoyancy has been previously reported (Fidalgo et al. 2006).

Buoyancy likely shortens life span by shifting cells from a semi-anaerobic state within yeast sediments to a highly oxygenated state while floating. Higher oxygen exposure implies increased levels of oxidized molecules and a reprogramming of the cellular redox homeostasis. We found hypoxic genes to be down-regulated in buoyant yeasts, while systems dealing with methionine oxidation and metabolism, in contrast, were broadly induced during the exponential phase. Nonoxidized methionine is essential for proper folding and function of proteins, synthesis of the central signaling molecule S-adenosine methionine, and the maintenance of glutathione pools, a key redox buffer (Brown-Borg and Buffenstein 2017). However, neither methionine supplementation nor restriction helped cells overcome the impact of buoyancy on life span. Because WA-*HPF1* cells both grew and expressed canonical oxidative stress responders normally, buoyancy impaired life span without causing detectable oxidative distress. One explanation is that cells experience very mild elevations in oxidation levels: enough to trigger a ROS signal but not enough to impose oxidative distress. Evidence does point to a role of oxidation in regulating cellular signaling that is partially disconnected from the handling of oxidative distress (Hanzén et al. 2016). For instance, upon exposure to a superoxide-generating agent, cells express 100-fold more Sod1p than is required for handling the associated distress, and this serves to promote Sod1p signaling through the yeast casein kinase Yck1p and through Sod1p translocating to the nucleus, where it serves as a transcription factor (Reddi and Culotta 2013; Tsang et al. 2014). At the end of the growth phase, cells sediment and become sedentary regardless of which *HPF1* allele they carry. Many yeast cells at this stage of their life cycle enter a quiescent state in which life span often is longer (Allen et al. 2006; Aragon et al. 2008; Davidson et al. 2011). WA-*HPF1* cells exhibit a reduced tendency to do so. Higher levels of superoxide anions impede entry into quiescence (Weinberger et al. 2010), and a mild elevation of superoxide anions could explain why WA-*HPF1* cells are less prone to enter quiescence. Increased methionine oxidation in WA-*HPF1* cells may be a downstream consequence of such a mild superoxide elevation, and an attractive speculation is that the methionine oxidation serves as the quiescence-inhibiting signal. This would explain why increasing the expression of the methionine reductase gene *MXR1* partially rescued the life span defect in WA-*HPF1* cells. Rapamycin supplementation, which restored *MXR1* expression upon chronological aging and rescued the short life span of WA-*HPF1* cells, in spite of these cells still being buoyant, could impinge directly on this superoxide/methionine oxidation signaling. TORC1 inhibits the expression of stress response genes, and its repression by rapamycin may prevent the increased methionine oxidation and allow WA-*HPF1* cells to enter quiescence normally (Fig. 5D). Furthermore, metabolomic analyses support extensive remodeling of methionine metabolism in aged flies (Avanesov et al. 2014), and the enzyme methionine sulfoxide reductase (Mxr) is a conserved modulator of the rate of aging in yeasts, flies, and mice (Moskovitz et al. 2001; Ruan et al. 2002; Kaya et al. 2010), suggesting that buoyancy-induced methionine oxidation in natural yeast strains impinges on a metabolic trait regulating aging in organisms from yeasts to mice.

*HPF1* and *FLO11*, like the vast majority of genes encoding cell wall proteins, contain intragenic tandem repeats (Verstrepen et al. 2005). Intragenic tandem repeats are dynamic in size, both due to strand-slippage during replication and ectopic recombination (Pâques et al. 1998; Fan and Chu 2007), and were found to fuel rapid yeast evolution (Verstrepen et al. 2005; Gemayel et al. 2012). Here, we showed that expansion of the N-terminal intragenic repeats within WA-*HPF1* was sufficient to shift yeasts toward a buoyant lifestyle, which reduced their life span. The majority (14/21) of amino acids in the expanded N-terminal repeat motif are serines or threonines, a huge overrepresentation compared to the 12% expected by their general prevalence in proteins (Kozlowski 2017). Serine and threonine are unique among amino acids in containing hydroxyl groups that directly facilitate hydrogen bonding with surrounding water molecules, an effect known to enhance the solubility of organic particles. An enticing possibility is therefore that the serine/threonine richness of the Hpf1 intragenic repeat expansion directly induces buoyancy. Furthermore, serine and threonine residues in cell wall and secreted proteins are highly O-glycosylated. Repeat expansion could thus increase cell wall glycosylation, which is predicted to improve solubility. The dynamic repeat expansions and contractions of *HPF1* may serve as a lifestyle switch, allowing rapid evolutionary shifts between buoyant and sedentary lifestyles in evolution, as dictated by fluctuating, opposing selection pressures. We note that the hydrophobic/hydrophilic properties of the yeast cell wall have been linked to buoyancy before (Palmieri et al. 1996; DeSousa et al. 2003; Fidalgo et al. 2006).

The WA-*HPF1* induced shift to a buoyant lifestyle in exponential phase, with a concomitant life span shortening, has no immediate parallels in higher multicellular organisms, such as humans. Nevertheless, stem cell fate and proliferation are determined by physical constraints and other environmental cues imposed by the surrounding extracellular matrix (Campisi 2001; Rando 2006; Guilak et al. 2009). For instance, fibroblast senescence can be reversed by culturing old cells in a young extracellular matrix (Choi et al. 2011). In addition, high oxygen exposure shortens the replicative life span of stem cells (Campisi 2001), while physiological levels of oxygen promote stem cell self-renewal (Topchiy et al. 2013; Sheshadri et al. 2015). Such cell-matrix interactions have been proposed to control human aging: the crosslinking theory of aging postulating that aging is a consequence of the progressive crosslinking of the extracellular matrix that impairs tissue homeostasis (Bjorksten 1968). It is quite likely that tandem repeats have a role in mediating these and other cell-environment interactions of relevance to human health and development. Intragenic tandem repeats occur in 17% of the genes in the human genome (Legendre et al. 2007) and are enriched in genes encoding extracellular proteins (Legendre et al. 2007; Gemayel et al. 2010). Repeat expansions in the transmembrane glycoprotein Muc1 improve tumor cell adhesion to lung tissue and thereby contributes to metastasis (Ciborowski and Finn 2002). Tandem repeat expansions have also been associated with human diseases, such as Huntington's chorea, Fragile X syndrome (Orr and Zoghbi 2007; Gemayel et al. 2010), and progeroid syndromes, such as myotonic dystrophy (Meinke et al. 2018). Besides controlling cell-extracellular matrix interactions, these tandem repeat variations tune human gene expression (Gymrek et al. 2016; Quilez et al. 2016), control nucleosome positioning (Volle and Delaney 2012), regulate circadian clocks (Sawyer et al. 1997), affect organismal morphology (Fondon and Garner 2004), and drive the evolution of pathogenic bacteria (Stern et al. 1986). It is now recognized that intragenic tandem repeat polymorphisms explain parts of the missing heritability that have evaded detection in genome-wide association studies (Hannan 2018; Gardiner et al. 2019). We expect that the ongoing development of long-read sequencing, which allows the rapid detection of intragenic repeat polymorphisms, will help illuminate their roles in many other classes of phenotypic variation, as illustrated here.

## Methods

### Strains

Phased Outbred Lines were derived from a cross between a North American oak tree strain (YPS128) and a West African palm wine strain (DBVPG6044) (Liti et al. 2009). Heterothallic (*ho::HygMX*) ancestral parents carrying *LYS2* or *URA3* at the *LYS2* locus (*lys2::URA3*) (Cubillos et al. 2009) were first mated to generate the NA/WA F1 hybrid (Supplemental Table S1). This F1 hybrid was used to generate a very large pool of progeny (10^6^–10^7^ cells) that was cycled through 12 rounds of alternating random mating, diploid selection, meiosis, sporulation, and haploid selection, resulting in a final pool of F12 outbred haploids (Parts et al. 2011). Eighty-six F12 haploid segregants of each mating type were randomly isolated from the outcrossed pool and sequenced, and their genotype was inferred using a set of 52,466 markers (Illingworth et al. 2013). The selected haploid F12 segregants genotypes allow systematic crossing (*MAT**a**, ura3::KanMX, ho::HygMX* paired with *MATα; ura3::KanMX; ho::HygMX; lys2::URA3*) to generate the prototroph POLs as described (Hallin et al. 2016) with minor modifications. F12 segregants were randomly paired and mated (in liquid YPD) in 96-well plates, and 1056 unique diploids with known, phased genomes were then selected during three consecutive diploid selective cultivations on liquid minimal media. Diploids were arrayed, stored, and analyzed in 96-well plates, each plate containing eight (three each of NA/NA and WA/WA, and two of NA/WA) internal controls used for life span normalization (Supplemental Table S1).

Reciprocal hemizygotes at the *HPF1* and *FLO11* loci were constructed in a NA/WA diploid hybrid, using genetically tractable NA and WA haploids, as described (Cubillos et al. 2009). Native *HPF1* and *FLO11* genes were deleted in haploids by homologous recombination with a NatMX4 cassette, using the lithium acetate/PEG transformation protocol (Cubillos et al. 2009), before being mated to the appropriate counterpart to generate diploids hemi- or homozygote for *HPF1* and *FLO11*. The same procedure was followed to build *FKH1*, *ASG1*, *RPL16A*, and *RPI1* reciprocal hemizygotes.

*HPF1* allele swapping was performed in two steps. First, part of *HPF1* (N-terminal repeats, C-terminal repeats, or internal part) was deleted in NA and WA haploids using homologous recombination with a *URA3* cassette. Then, the *HPF1* segments to be swapped were PCR-amplified from the desired alleles with Platinum SuperFi (Thermo Fisher Scientific) DNA polymerase and swapped into the orthologous position of the recipient strain using homologous recombination (targeting identical nonrepeated sequences for both alleles) and selected on 5-FOA. Strains obtained were then mated to *hpf1*::NatMX4 haploids to generate the indicated diploids. The same procedure was followed to perform *FLO11* allele swapping. A summary of strains and primers used in this study can be found in Supplemental Tables S1 and S2, respectively.

### Media

YPD (1% yeast extract, 2% peptone, 2% dextrose, 2% agar [MP Biomedicals]) was used for all matings. Mated cells were streaked on synthetic minimal medium (2% dextrose [Sigma-Aldrich], 0.675% yeast nitrogen bases [Formedium], pH set to 6.0 with 2.5 M NaOH) to select for diploids. Counter selection of *URA3* cells for allele swapping was made on 5-FOA plates (2% dextrose [Sigma-Aldrich], 0.675% yeast nitrogen base [Formedium], 0.088% uracil drop-out [Formedium], 0.005% uracil [Sigma-Aldrich], 2% agar, 0.1% 5-FOA [Sigma-Aldrich]). Pseudohyphal growth was induced on SLAHD plates (2% dextrose [Sigma-Aldrich], 1.7 g/L yeast nitrogen base without ammonium sulfate [Formedium], 6.6 mg/L ammonium sulfate [Formedium], 42 mg/L histidine [Sigma-Aldrich], 2% agar) (Hope and Dunham 2014).

Life span was estimated in: (1) calorie-rich synthetic dextrose complete (SDC) media (2% dextrose, 0.675% yeast nitrogen base [Formedium], 0.088% complete amino acid supplement [Formedium], pH set to 6.0 with 2.5 M NaOH); (2) calorie-restricted media (SDC as above, but with 0.5% dextrose instead of 2%) (Jiang 2000; Lin et al. 2000; Smith et al. 2007); and (3) rapamycin supplemented media (SDC supplemented with 0.025 µg/mL rapamycin [Sigma-Aldrich]) (Vázquez-García et al. 2017; Li et al. 2019). Methionine supplementation was performed in SDC supplemented with 500 mg/L methionine (15× Met), while methionine restriction was performed in SDC completely deprived of methionine (0× Met, complete amino acid supplement substituted with methionine drop-out). Oleic acid supplementation was performed in SDC supplemented with 0.1% oleic acid.

### Chronological life span assay

Cells cultivated overnight in calorie-rich (SDC) media were diluted (1:100) in 200 µL of either fresh SDC, or CR, or RM media in a 96-well plate. Cultivation plates were sealed with adhesive aluminum foil to prevent evaporation and incubated at 30°C. Aging was considered to start at saturation of the culture, 72 h post-inoculation (Fabrizio and Longo 2007), and cells were kept in saturated media for the whole duration of the experiment unless otherwise specified. To generate hypoxia, overnight SDC cultures were diluted (1:100) and aged in different volumes (3, 15, 30, or 50 mL) of fresh SDC in static sealed FALCON tubes. When CLS was performed in water, 72-h stationary cultures were centrifuged, and cells were washed 3× before being resuspended and kept in the same volume of distilled water. For oleic acid and methionine supplementation or restriction, overnight SDC cultures were diluted (1:100) and aged in 200 µL of the appropriate SDC-based media (see Media subsection).

Aging was measured as viable cells (%) by flow cytometry based on the uptake of the fluorescent molecules propidium iodide (PI) and YO-PRO-1 iodide (YP). Propidium iodide and YO-PRO-1 are membrane-impermeable nucleic acid binding molecules that enter into necrotic but not into alive cells. Therefore, nonfluorescent cells are alive, while fluorescent cells are not. YO-PRO-1 penetrates also into apoptotic cells (Herker et al. 2004; Wlodkowic et al. 2009). At each aging time point (7, 21, 35 d after entry into quiescence unless otherwise stated), 5 µL of cells were transferred into 100 µL of staining solution (phosphate buffer saline + 3 µM propidium iodide [Sigma-Aldrich] + 200 nM YP [Thermo Fisher Scientific]) in a 96-well plate and incubated for 5 min in the dark at 30°C. The samples were analyzed on a FACS-Calibur flow cytometer (Becton Dickinson) using a High Throughput Sampler (Becton Dickinson) device to process 96-well plates and detect fluorescence with FL-1 (YP) and FL-3 (PI) channels. POLs experiments, where each allele is present and therefore replicated in many lineages, were run in single replicate, and viability estimates were normalized to those of eight internal controls run on the same plate. All other experiments were run at least in triplicate.

### Linkage analysis

Linkage analysis was performed as described (Hallin et al. 2016). Briefly, mapping of life span QTLs was done using the normalized POL life spans, the R/qtl package in R (Broman et al. 2003), and the marker regression method in the scanone function. Significance thresholds were calculated with 1000 permutations to call QTLs with a significance level of 0.05. Confidence intervals for the peaks were calculated using a 1.8-LOD drop using the lodint function in R/qtl. We corrected for population structure by using the deviation of the life span of each POL from the parent mean.

### Fraction and viability of quiescent cells

Isolation of quiescent cells was performed as described (Allen et al. 2006) with minor modifications. Cells were grown overnight in SDC and 1:100 diluted into 200 µL of fresh SDC in a 96-well plate. Each strain was distributed 96 times in the same 96-well plate and incubated for either 3 d or 10 d (1 strain per 96-well plate per time point). At the indicated time point, the 96 replicates of each strain were collected in a single FALCON tube, washed once with Tris buffer (50 mM, pH 8), and resuspended in 1 mL Tris buffer. Cells were then gently overlaid on the top of the preformed Percoll gradient (8 mL) and centrifuged at 400*g* at 20°C for 1 h. Upper and lower phases were equally split using a ruler and isolated by pipetting in distinct FALCON tubes. Ten milliliters of PBS were added into each tube prior to vortexing to homogeneously resuspend the cells. Two microliters of each tube were sampled and mixed with 100 µL of PI/YP staining solution, and viability was determined by cytometry as described in the chronological life span assay above. Cell concentration was given by the cytometer and used to calculate the ratio of quiescent/nonquiescent cells. The whole experiment was run with two independent replicates for each strain.

### RNA-seq

We extracted RNA from SDC- and RM-cultivated cells in exponential phase and after 7 d of aging using the KAPA Biosystems Hyperplus kit with RiboErase, as per the manufacturer's instructions at 500 ng input. RNA integrity was assessed on an Agilent Bioanalyzer using the RNA Pico kit to determine RIN scores. Library quality was assessed using an Agilent Bioanalyzer using the DNA 1000 kit. Libraries were quantified using a KAPA Biosystems library quantification kit. The libraries were normalized, pooled, and loaded onto a NextSeq 500/550 High Output v2 kit (300 cycles) flow cell and sequenced on a NextSeq 500 instrument.

We performed transcript-level abundance quantification by pseudo-aligning RNA-seq reads to the coding sequences of a *Saccharomyces* Genome Database (SGD) yeast reference gene set, using kallisto (v0.44.0) (Bray et al. 2016). In this way, we obtained the transcripts per kilobase million (TPM) value for each gene in each sample as its normalized expression level, which is directly comparable, both among different genes and among different samples. Sleuth (0.30.0) (Pimentel et al. 2017) was further used to assess the statistical difference of the same gene between different samples by decoupling biological differences from experimental noise. False discovery rate (FDR; α = 0.05) adjustment (Benjamini and Hochberg 1995) was further applied for multiple test correction.

Total RNA was extracted from two biological replicates either during exponential growth or after 7 d of aging in SDC or in RM. Global expression level was analyzed by pairwise comparison of the NA/WA hybrids hemizygotes for WA and NA-*HPF1* within each environment and time point to identify differentially expressed transcripts. Standard GO term analysis was performed on greater than twofold differentially expressed genes with the GO Term Finder tool available at SGD, with an FDR corrected α threshold of 0.01.

### CRISPR-Cas9-mediated genomic insertion

The *MXR1, SOD1*, and *CTT1* open reading frames were first cloned in frame with the *TEF1* promoter and *CYC1* terminator using BamHI and EcoRI restriction. The overexpression cassettes were then amplified with the primers 1492 and 1461 (Supplemental Table S2), containing 50-bp homology to target the *HO* locus, and 30-bp and 23-bp homology with TEF1p and CYC1t, respectively. The PCR product was provided as donor DNA. Genomic integration was mediated by CRISPR-Cas9 based on the pUDP004 single gRNA and Cas9 system (Addgene no. 101165) (Gorter de Vries et al. 2017). The amidase selection marker was swapped for nourseothricin (Nat) for antibiotic resistance by Gibson assembly using NEBuilder HiFi DNA Assembly of the amplified pUDP004 backbone, yielding the pL59-Nat plasmid. Two highly conserved gRNA sites in the *HO* gene were identified—gRNA1: TATTTTTATAAAGATTGGAG and gRNA2: TAAAGACATCGCAAACGTCA, 100% identical in all *S. cerevisiae* and *S. paradoxus* strains in the yeast population reference panel (Yue et al. 2017). These gRNAs were inserted into the BsaI-cut pL59-Nat plasmid by Gibson assembly of two synthesized fragments containing gRNA1 and gRNA2, respectively, yielding the pL90-Nat-2×HO plasmid. The reverse complement of the first six bases of the gRNA were included for splicing with the hammerhead ribozyme. For CRISPR-Cas9 editing, 500 ng of pL90-Nat-2×HO and 50 µL of donor DNA PCR reactions were transformed following the lithium acetate/PEG protocol (Cubillos et al. 2009). pL59-Nat and the pL90-Nat-2×HO were submitted to Addgene (139069 and 140465, respectively).

### Pseudohyphal growth and surface adhesion

For pseudohyphal growth, yeasts were grown overnight in YPD, and single cells were dropped at precise locations on a SLAHD plate (see Media subsection) by micromanipulation. Cells were incubated at 30°C for 24 h before acquiring pictures. For surface adhesion, 10 µL of YPD overnight culture were dropped on a YPD plate and incubated at 30°C. After 6 d, yeast patches were washed with sterile water and smoothly rubbed with a finger.

### Growth rate

Cells were grown overnight in SDC before being diluted 1:100 into 200 µL of fresh SDC in a 96-well plate and incubated at 30°C without shaking. Every 2 h, cells were resuspended by pipetting, and the optical density (600 nm) was measured using a Tecan Infinite M200 plate reader.

### Software

Data analysis, plots, and statistical tests were performed with R (R Core Team 2019) and RStudio (https://www.rstudio.com). Figures were prepared using Adobe Illustrator CC and Adobe Photoshop CC. Genetic engineering, molecular cloning, and self-alignment of *HPF1* and *FLO11* were designed using Geneious R9.1.8 (https://www.geneious.com).

## Data access

Raw RNA-seq data from this study have been submitted to the NCBI BioProject database (https://www.ncbi.nlm.nih.gov/bioproject) under accession number PRJNA544860.

## Competing interest statement

The authors declare no competing interests.

## Supplementary Material

Supplemental Material
