# Supplementary material for: Intragenic repeat expansion in the cell wall protein gene HPF1 controls yeast chronological aging
Source: Genome Res. 2020 May;30(5):697–710. doi: 10.1101/gr.253351.119 (PMC7263189; doi:10.1101/gr.253351.119)
Supplement: Supplemental Material [file supp_30_5_697__index.html]

Intragenic repeat expansion in the cell wall protein gene HPF1 controls yeast chronological aging — Supplemental Material 

# Intragenic repeat expansion in the cell wall protein gene *HPF1* controls yeast chronological aging

## Supplemental Material

- Supplemental\_Figures\_S1-S8.pdf
- Supplemental\_Table\_S1.xlsx
- Supplemental\_Table\_S2.xlsx
- Supplemental\_Table\_S3.xlsx
- Supplemental\_Table\_S4.xlsx
- Supplemental\_Table\_S5.xlsx
- Supplemental\_Table\_S6.docx
