# Supplementary material for: Intragenic repeat expansion in the cell wall protein gene HPF1 controls yeast chronological aging
Source: Genome Res. 2020 May;30(5):697–710. doi: 10.1101/gr.253351.119 (PMC7263189; doi:10.1101/gr.253351.119)
Supplement: Supplemental Material [file supp_gr.253351.119_Supplemental_Figures_S1-S8.pdf]

SUPPLEMENTAL FIGURES

Intragenic repeat expansion in the cell wall protein gene *HPF1* controls yeast chronological aging

Benjamin P Barré[1*], Johan Hallin[1], Jia-Xing Yue[1], Karl Persson[2], Ekaterina Mikhalev[3], Agurtzane Irizar[1], Sylvester Holt[1], Dawn Thompson[3], Mikael Molin[4], Jonas Warringer[2] and Gianni Liti[1*]

[1]Université Côte d'Azur, CNRS, INSERM, IRCAN, Nice, France; [2]Department of Chemistry and Molecular Biology, University of Gothenburg, Gothenburg, Sweden; [3]Ginkgo Bioworks Inc. Boston, MA 02210, USA; [4]Department of Biology and Biological engineering, Chalmers University of Technology, Gothenburg, Sweden

*Correspondence should be addressed to B.B. (byngeamain@gmail.com) or to G.L. (gianni.liti@unice.fr).

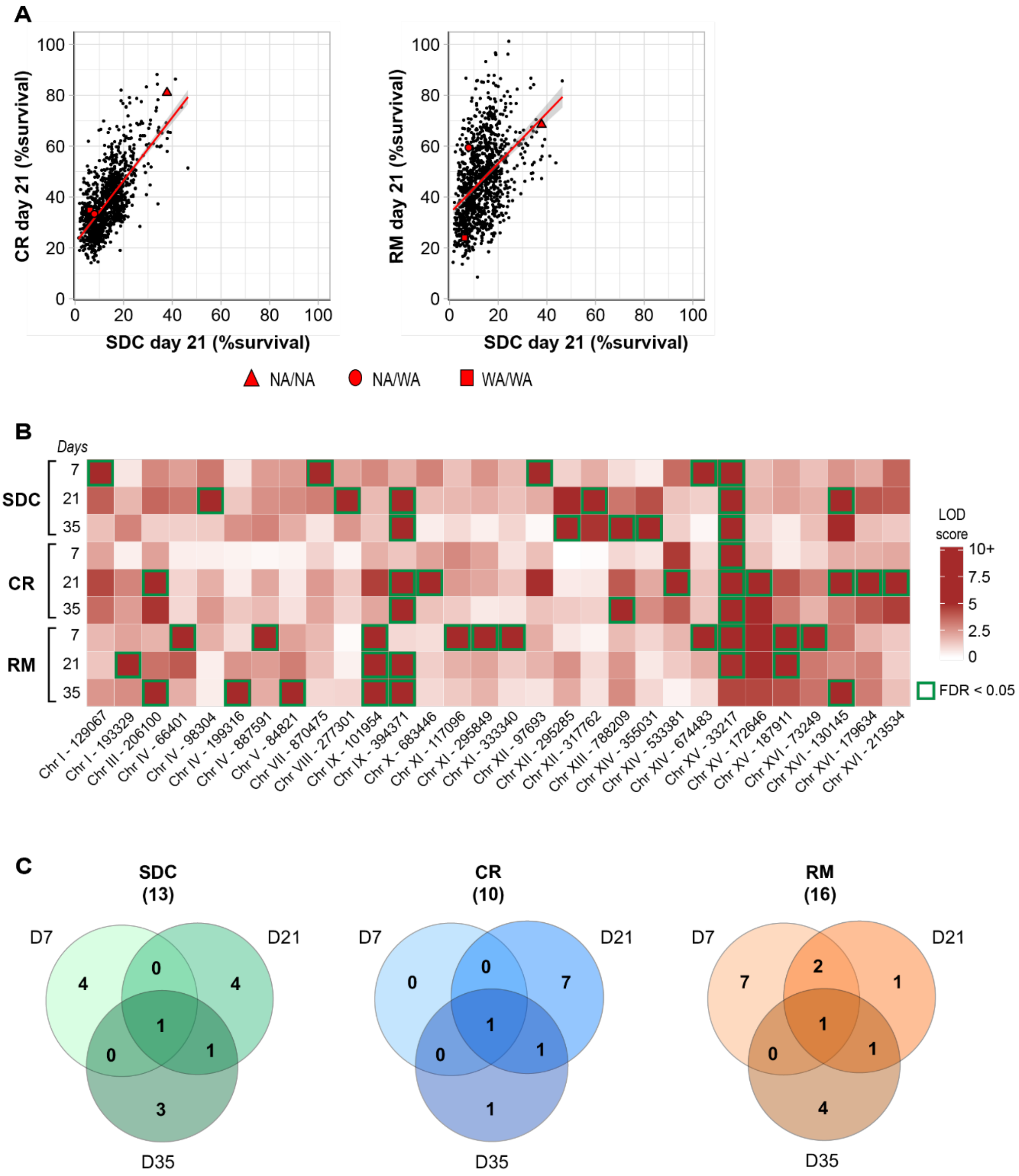

Figure S1. Genetic, environmental and time variation in chronological life span
(A) Scatter plots of CLS of selected environments at 21 days for the 1056 POLs (reported in Fig. 1A) underlie CLS correlation across conditions. Parental strains and F1 hybrid are indicated with red symbols. Linear regression is represented as a red line with 95% confidence interval. **(B)** Summary heat map of the 30 distinct QTL regions that were mapped. Green frames indicate conditions for which QTLs were significant (FDR < 0.05). **(C)** Venn diagram summarizing the distribution of QTLs across time points for each environment. D7, D21, and D35 refers to 7, 21, and 35 days of chronological aging, respectively. The value in parenthesis indicates the total number of QTLs found per environment.

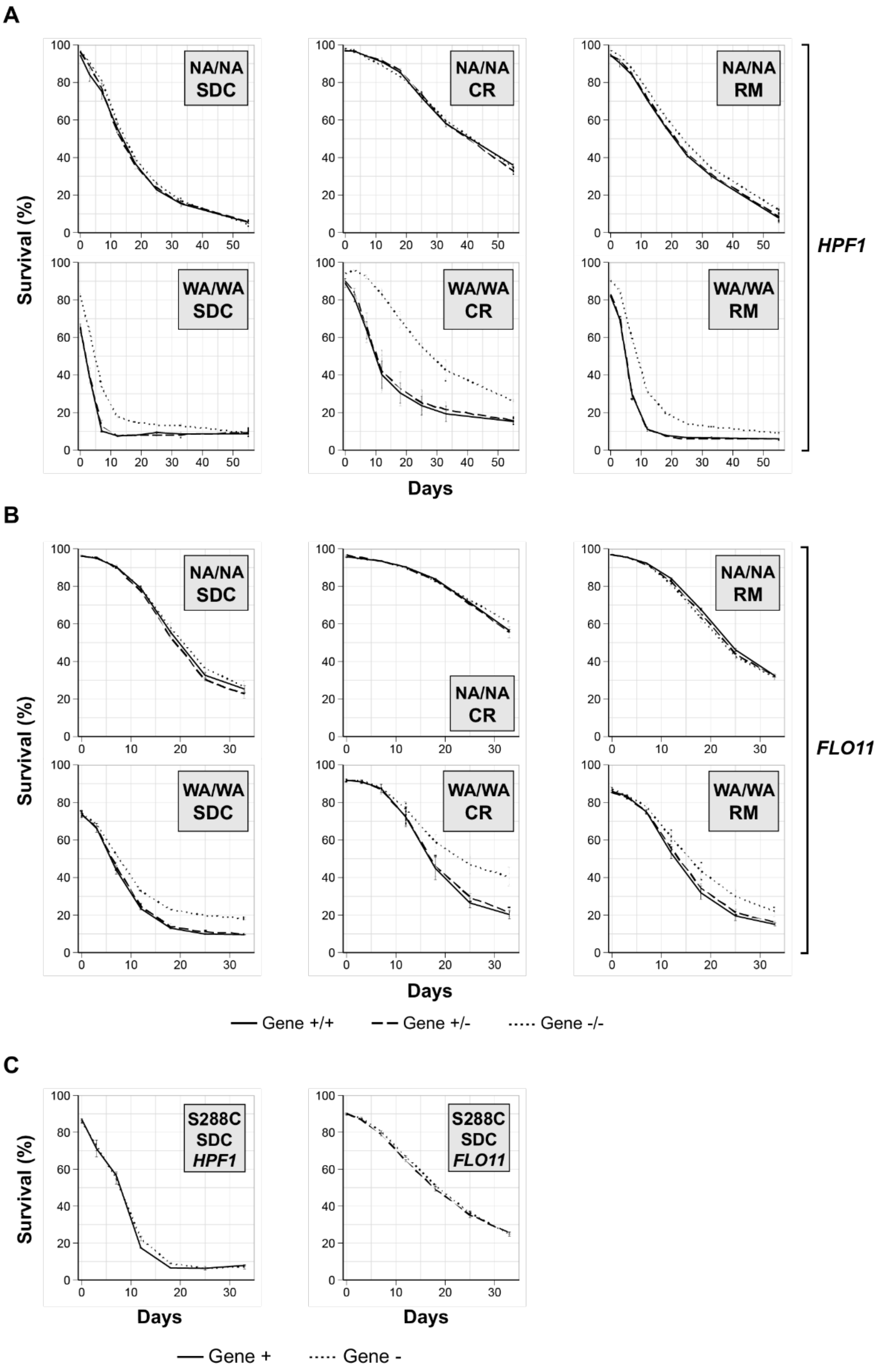

Figure S2. Effect of *HPF1* and *FLO11* deletions on homozygote parental strains and lab strain S288C
(A) Effect of *HPF1* deletion on NA and WA homozygote parents. Strain background and environmental condition are indicated in grey boxes. Solid lines, wild-type; dashed lines, one copy deleted; dotted lines, complete deletion. **(B)** Effect of *FLO11* deletion on NA and WA homozygote parents (see above). **(C)** Effect of *HPF1* and *FLO11* deletion on the lab strain S288C in SDC. Gene deleted is indicated in the grey box. Solid lines, wild-type; dotted lines, complete deletion.

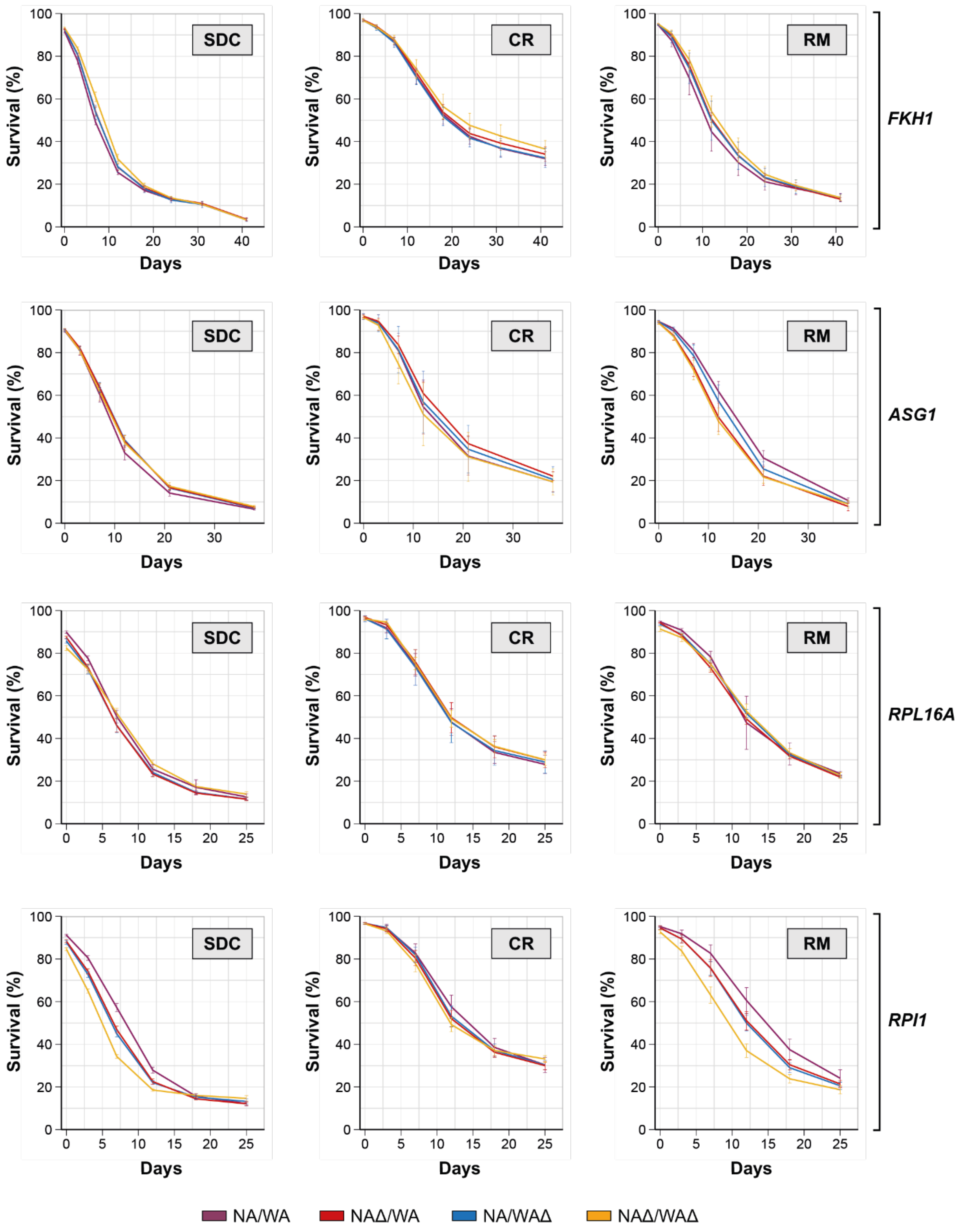

Figure S3. Reciprocal hemizygote analysis of candidate genes at Chr IX rapamycin specific QTL
Reciprocal hemizygosity. CLS of NA/WA hemizygotes (blue: WAΔ; and red: NAΔ), heterozygote (purple: NA/WA) and complete deletion (yellow; NAΔ/WAΔ) of either *FKH1*, *ASG1*, *RPL16A* or *RPI1*.

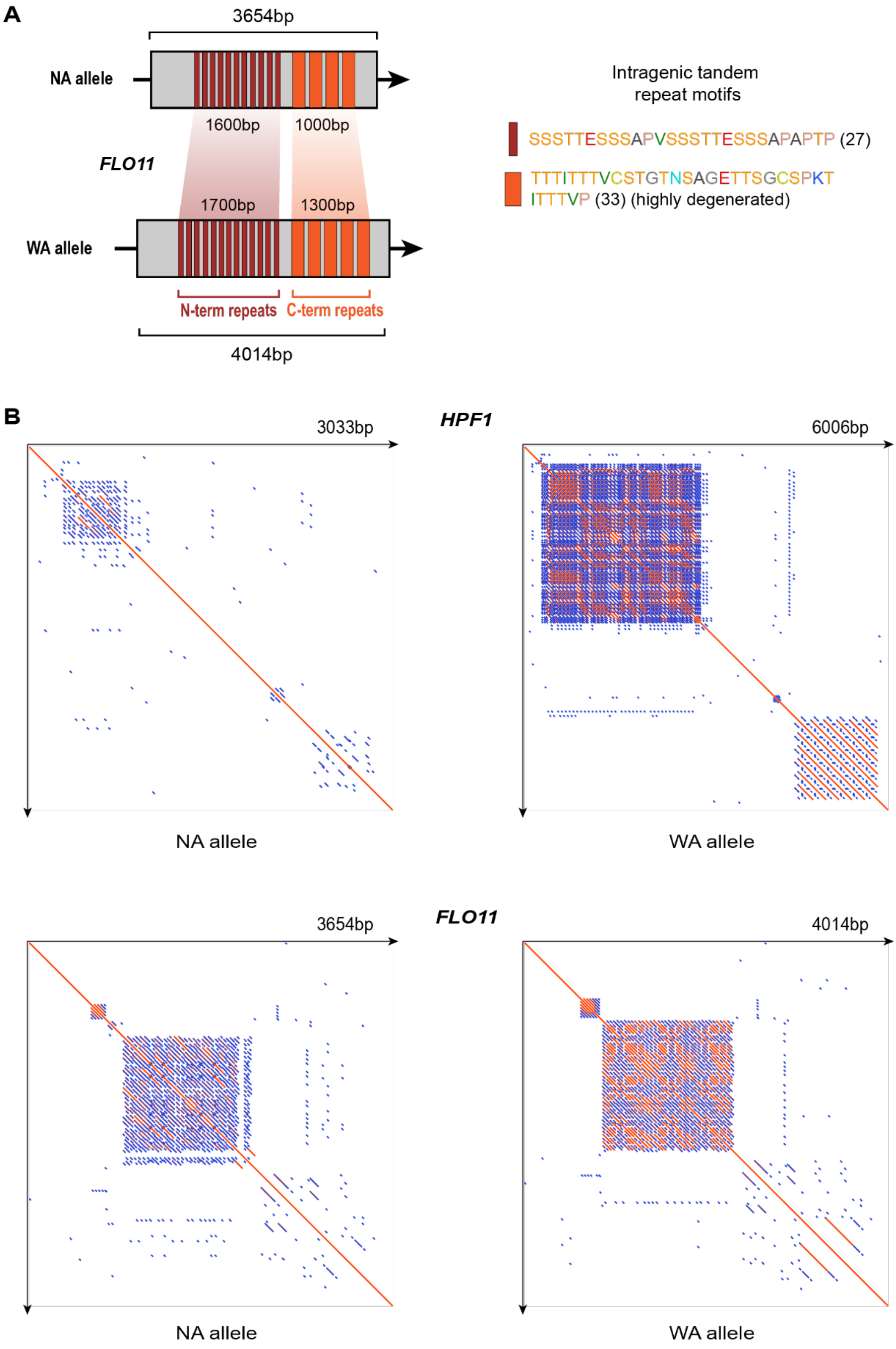
A
3654bp
NA allele
1600bp
1000bp
FLO11
1700bp
1300bp
WA allele
N-term repeats
C-term repeats
4014bp
Intragenic tandem repeat motifs
SSSTTESSSAPVSSSTTESSSAPAPTP (27)
TTTITTTVCSTGTNSAGETTSGCSPKT ITTTVP (33) (highly degenerated)
B
3033bp
HPF1
6006bp
NA allele
WA allele
3654bp
FLO11
4014bp
NA allele
WA allele

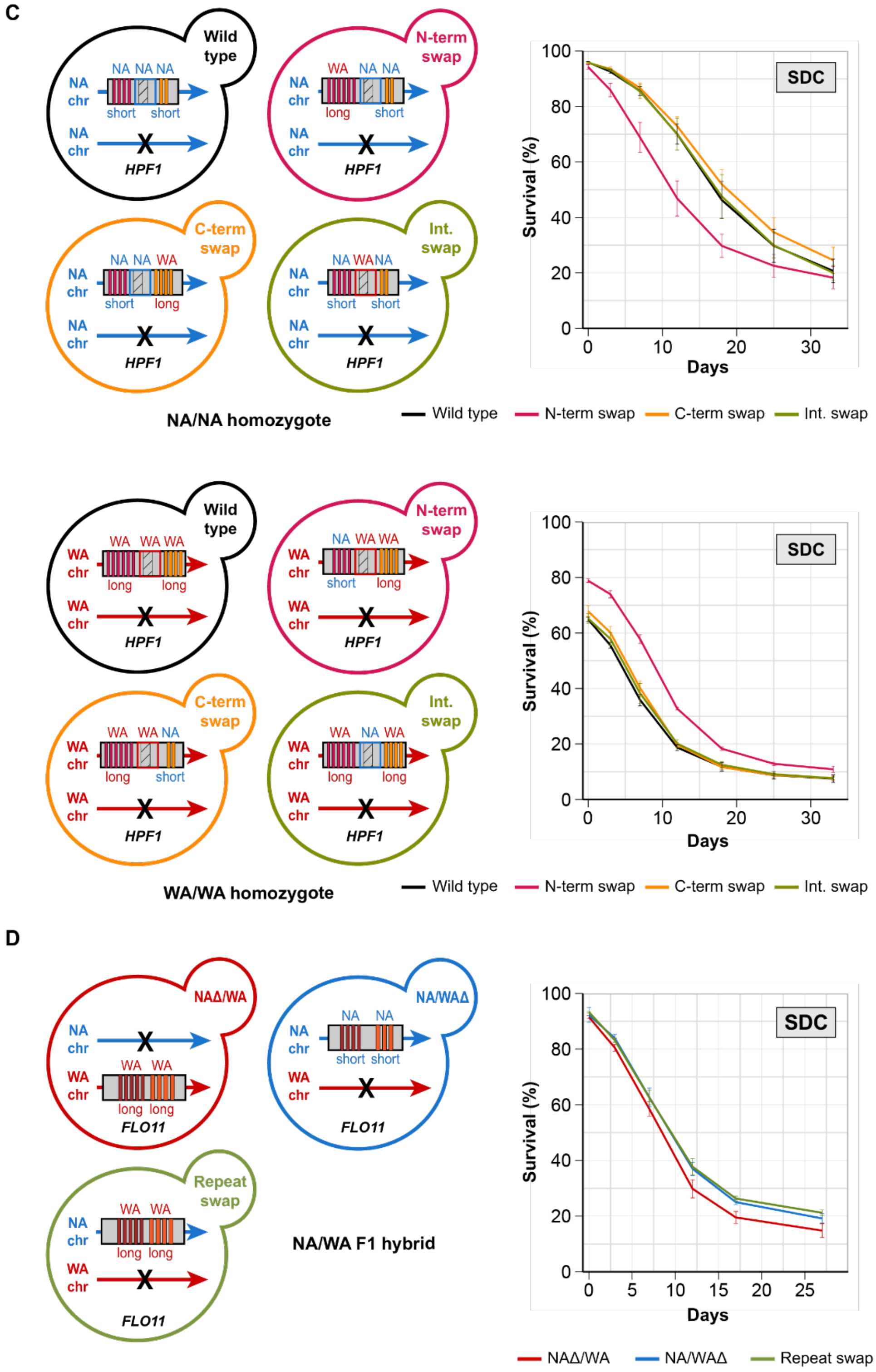

Figure S4. Repeat expansion within *FLO11* and *HPF1*

(A) Schematic representation of the intragenic repeats (coloured rectangles) in *FLO11* for NA and WA alleles. Amino acids are colored according to the RasMol nomenclature. Numbers = motif size (amino acids). **(B)** Self-alignment of *HPF1* (top) and *FLO11* (bottom) ORFs for the NA (left) or WA alleles (right) generated with Geneious. **(C)** Allele swapping of different *HPF1* parts effect on CLS. The NA/NA (top) and WA/WA (bottom) homozygotes were engineered as schematically represented on the left. **(D)** Allele swaps of *FLO11* intragenic tandem repeats in the NA/WA F1 hybrid. The NA-*FLO11* tandem repeats were replaced by their WA-*FLO11* counterpart (green). Right panel: CLS of the allele swapped construct (green) compared to *FLO11* reciprocal hemizygotes (red: NAΔ/WA; and blue: NA/WAΔ) in SDC media.

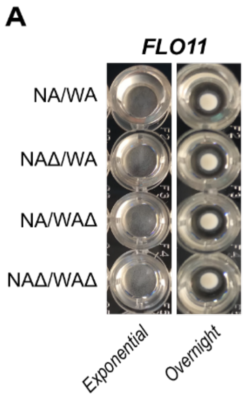

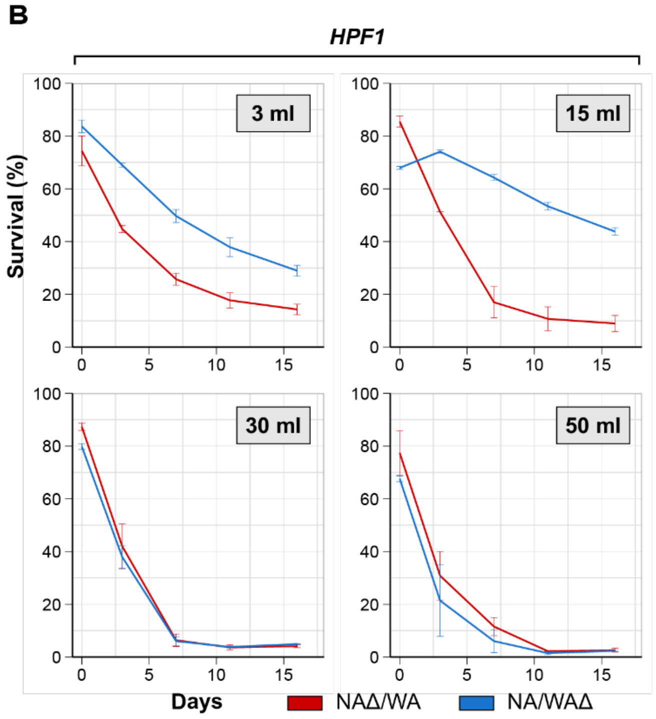

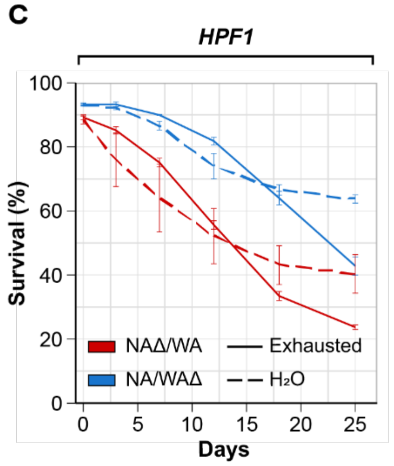

D

HPF1

Timepoint	SDC		CR		RM	
	NAΔ/WA	NA/WAΔ	NAΔ/WA	NA/WAΔ	NAΔ/WA	NA/WAΔ
Inoculation	6.00	6.00	6.29	6.29	6.00	6.00
Mid-Exponential	3.98	4.10	4.54	4.71	3.97	4.04
7 days	3.52	3.53	4.69	4.68	3.47	3.50
21 days	3.50	3.51	4.63	4.88	3.44	3.41

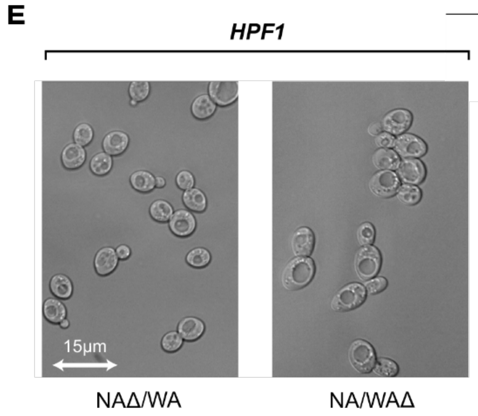

Figure S5. Buoyancy effect on life span

(A) Buoyancy is not affected by *FLO11* allelic variation. Cells were cultivated for 7 hours (exponential phase) or overnight in calorie rich (SDC) medium in a 96-well plate. **(B)** CLS of *HPF1* reciprocal hemizygotes incubated in static tubes filled with increasing volumes of SDC medium as indicated in the grey boxes. **(C)** CLS of *HPF1* reciprocal hemizygote strains in water (Δ indicates which *HPF1* allele was deleted). Cells were pre-grown 3 days in SDC before being washed and resuspended in water to rule out any possible secretion and acidity effects or kept in exhausted media that would contain secreted Hpf1p. Error bars represent standard deviations. **(D)** pH of *HPF1* reciprocal hemizygote cultures during CLS. Cells were grown and aged either in SDC, CR, or RM, as indicated. Inoculation refers to the pH at the time of initial incubation, mid-exponential corresponds to 7 hours post-inoculation. **(E)** Microscopy images of *HPF1* reciprocal hemizygotes during exponential growth in calorie rich SDC medium.

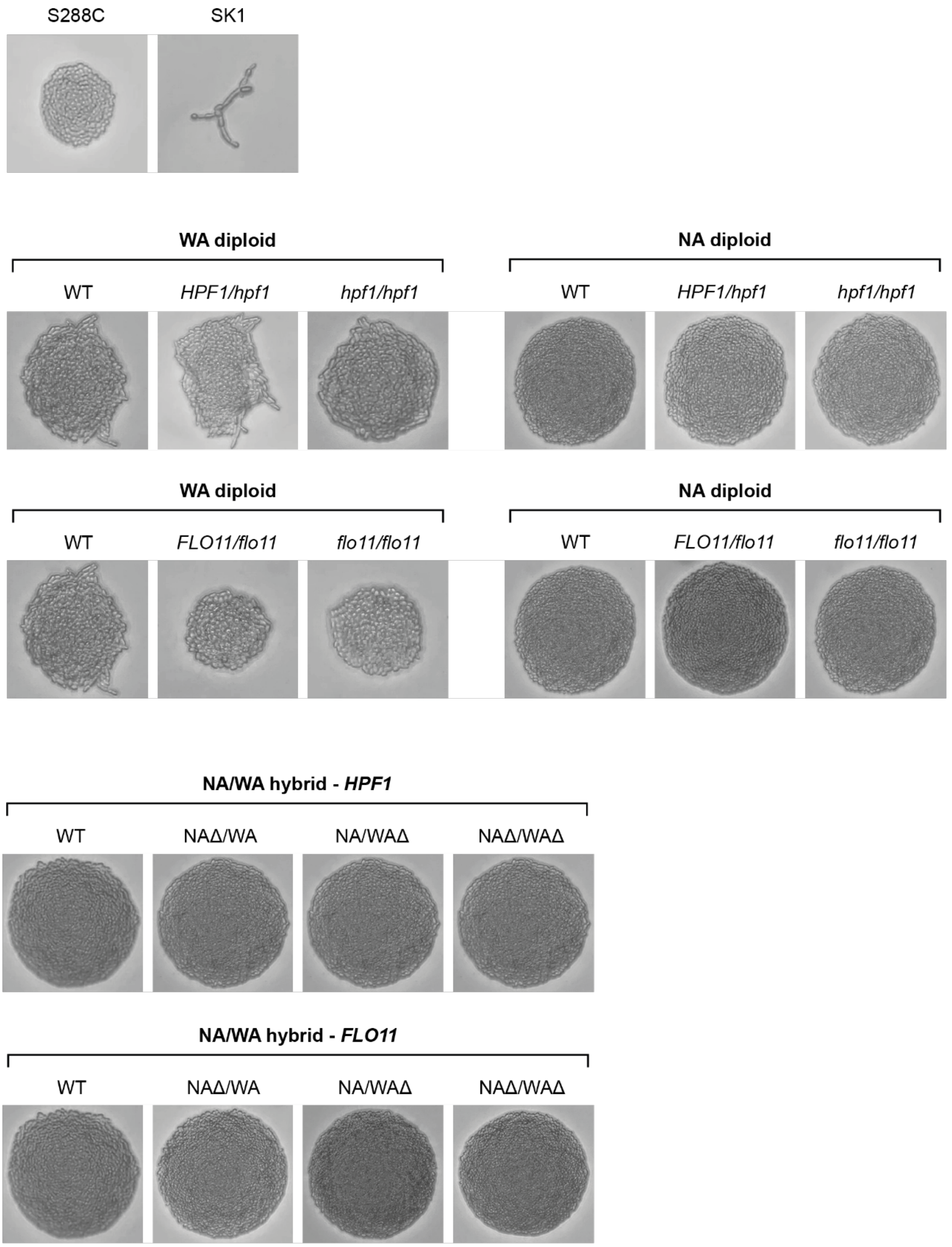

Figure S6. Pseudohyphal growth does not mediate *HPF1* and *FLO11* CLS effects
Pseudohyphal growth of the lab strains S288C and SK1 (top), NA and WA homozygotes with or without *HPF1* or *FLO11* deletion (middle), and *HPF1* or *FLO11* reciprocal hemizygotes (bottom) grown on SLAHD plates.

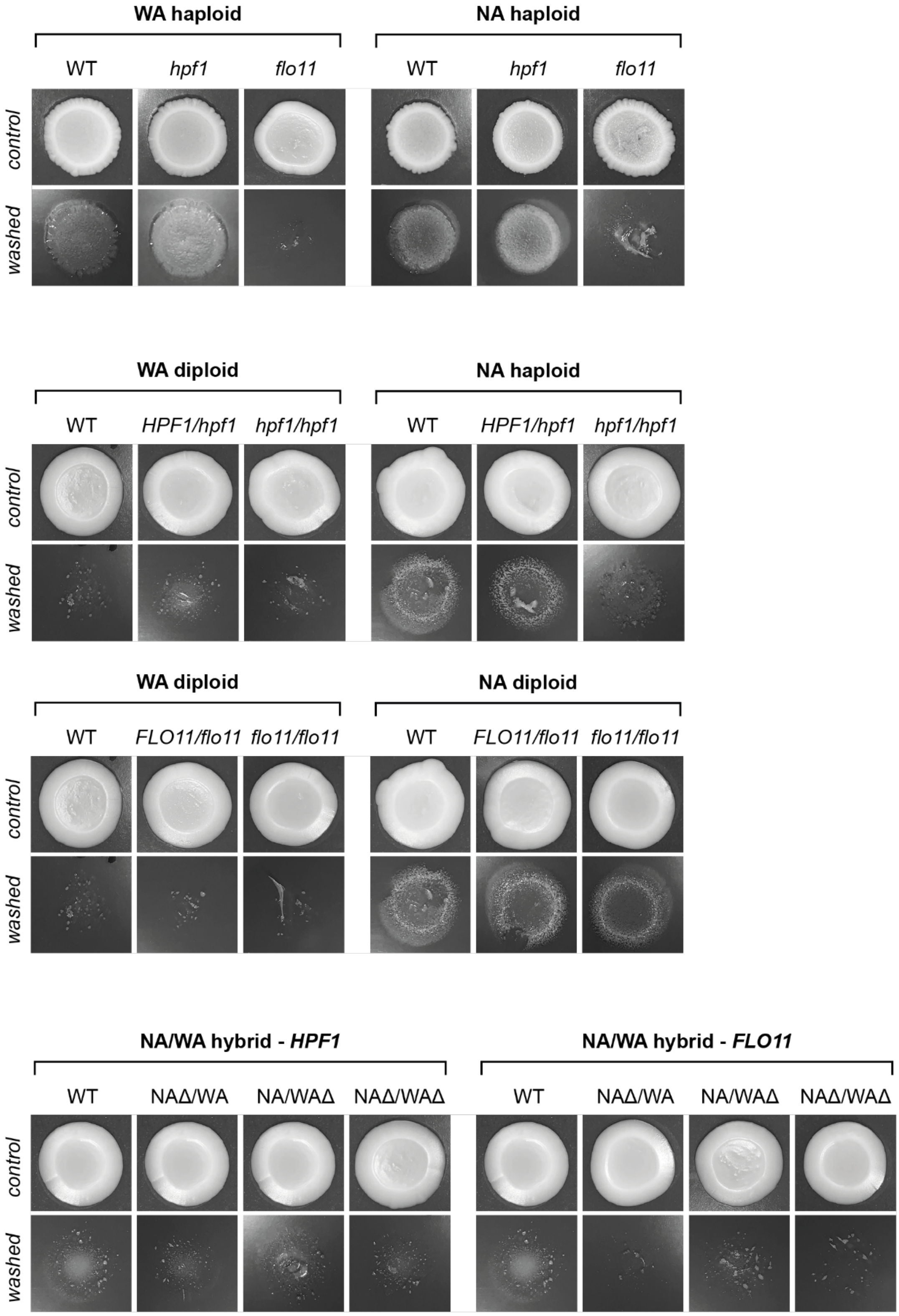

Figure S7. Adhesion does not mediate *HPF1* and *FLO11* CLS effects

Cell-surface adhesion capacity of NA and WA haploids (top) and homozygotes (middle) with or without *HPF1* or *FLO11* deletion, and of *HPF1* or *FLO11* reciprocal hemizygotes (bottom). Cells were grown 6 days on YPD plates and photographed before (control) and after (washed) washing with water.

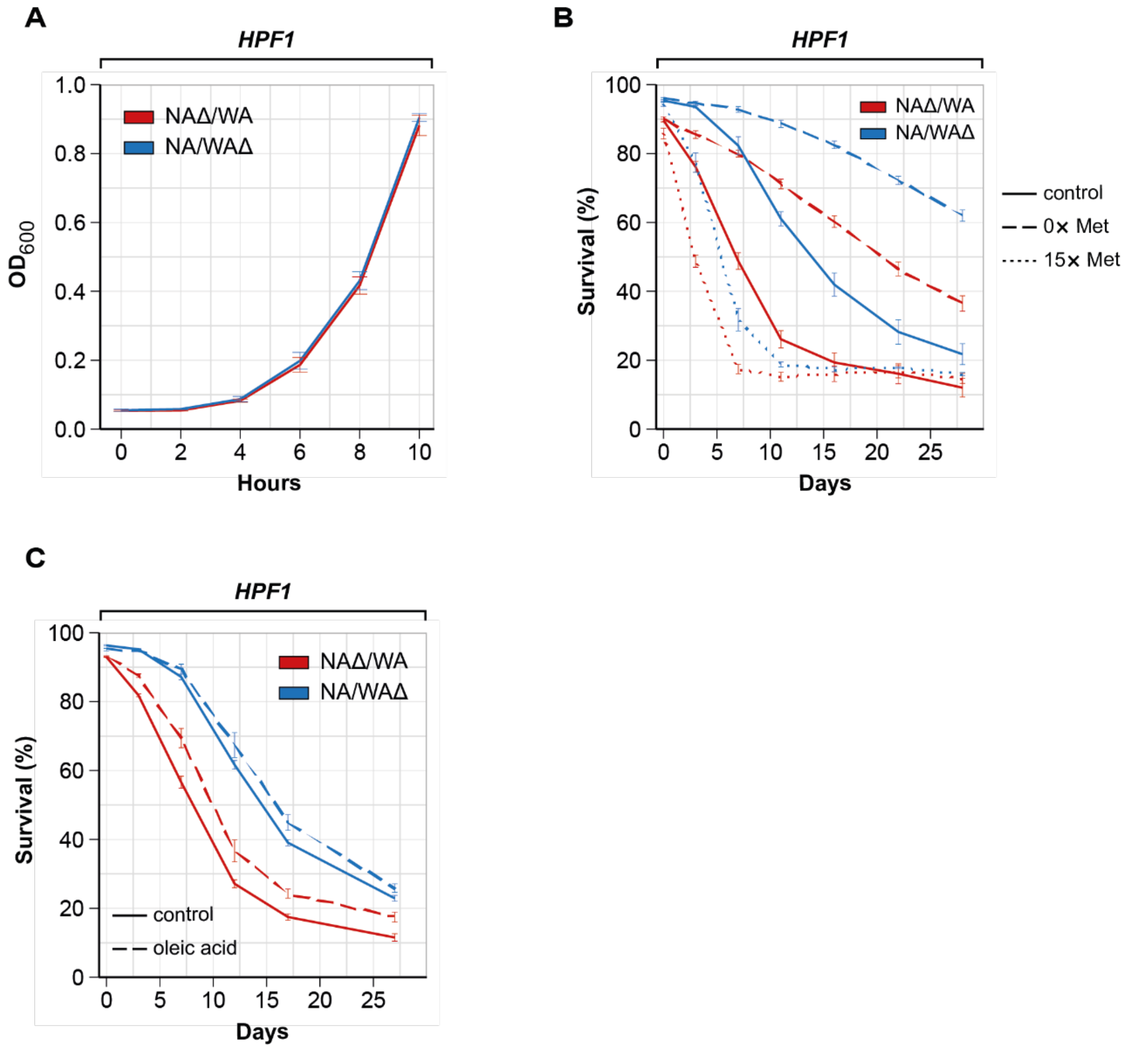

Figure S8. Growth in SDC and CLS in methionine and oleic acid regimens

(A) Growth rate of *HPF1* reciprocal hemizygotes in SDC medium. Cells were grown in static 96-well plates and resuspended by pipetting before measuring optical density at 600 nm. **(B-C)** Effect of methionine restriction and supplementation (B), and of oleic acid supplementation (C), on the CLS of *HPF1* reciprocal hemizygotes. Cells were incubated and aged in the same SDC-based methionine deprived (no methionine; 0×), methionine supplemented (15 times control concentration; 15×), or oleic acid supplemented (0.1%) media.
