# Supplementary material for: Intragenic repeat expansion in the cell wall protein gene HPF1 controls yeast chronological aging
Source: Genome Res. 2020 May;30(5):697–710. doi: 10.1101/gr.253351.119 (PMC7263189; doi:10.1101/gr.253351.119)
Supplement: Supplemental Material [file supp_gr.253351.119_Supplemental_Table_S6.docx]

**TABLE S6 – *FLO11* and *HPF1* DNA sequences (ORF)**

| **>*FLO11*_DBVPG6044** |
| --- |
| ATGCAAAGACCATTTCTACTCGCTTATTTGGTCCTTTCGCTTCTATTTAACTCAGCTTTGGGTTTTCCAACTGCACTAGTTCCTAGATGCTCCGAAGGAACTAGCTGTAATTCTATCGTTAATGGCTGTCCCAACTTAGACTTCAATTGGCACATGGACCAACAGAACATCATGGAGTATACTTTGGATGTGACTTCTGTTTCTTGGGTTCAAGACAACACGTACCAAATCACTGTTCATGTCAAAGGTAAAGAAAACATTGACCTAAAATATCTATGGTCTTTGAAAATCATTGGTGTTACTGGTCCAAAAGGTACCGTCCAACTATACGGTTACAACGAAAATACCTATTTGATTGACAACCCAACTGATTTCACAGCCACTTTTGAAGTCTATGCCACACAAGATGTCAACAGCTGTCAGGTGTGGATGCCTAACTTCCAAATTCAATTCGAGTATTTGCAAGGTAGTGCCGCTCAATATGCAAGCTCTTGGAAATGGGGAACTACATCTTTTGATTTGTCTACTGGTTGTAACAACTATGACAATCAAGGCCACTCTCAAACAGATTTCCCAGGCTTCTATTGGAACATAGATTGTGACAACAATTGTGGCGGTACGAAGTCATCTACCACTACATCAACTAGTACTTCCGAGTCATCTACCACTACATCAACTAGTACTTCCGAGTCATCTACCACTACATCAACTAGTACTTCCGAGTCATCTACCACTACATCAACTAGTACTTCCGAGTCATCTACCACTACATCTAGCACTTCCGAGTCATCTACCACTACATCAACTACCACTTCAGAGTCATCTACATCATCATCAACCACCGCTCCTGCTACACCAACCACTACCGAAAGCTCTTCTGCTCCAGTACCAACTCCATCAAGCTCTACTACTGAAAGCTCTTCTGCTCCAGTAACCAGCTCCACCACTGAAAGCTCTTCTGCTCCAGTAACCAGCTCCACCACTGAAAGCTCTTCTGCTCCAGCTCCAACTCCATCCAGCTCTACTACCGAAAGCTCTTCTGCTCCAGTATCCAGCTCCACCACTGAAAGCTCTTCTGCTCCAGCTCCAACTCCATCCAGCTCTACTACCGAAAGCTCTTCTGCTCCAGTAACCAGCTCCACCACTGAAAGCTCTTCTGCTCCAGTAACCAGCTCCACCACTGAAAGCTCTTCTGCTCCAGTAACCAGCTCCACCACTGAAAGCTCTTCTGCTCCAGTACCAACTCCATCAAGCTCTACTACTGAAAGCTCTTCTGCTCCAGTAACCAGCTCCACCACTGAAAGCTCTTCTGCTCCAGTACCAACTCCATCAAGCTCTACTACTGAAAGCTCTTCTGCTCCAGTACCAACTCCATCAAGCTCTACTACTGAAAGCTCTTCTGCTCCAGTACCAACTCCATCCAGCTCTACTACCGAAAGCTCTTCTGCTCCAGTACCAACTCCATCAAGCTCTACTACTGAAAGCTCTTCTGCTCCAGTAACCAGCTCCACCACTGAAAGCTCTTCTGCTCCAGTAACCAGCTCCACCACTGAAAGCTCTTCTGCTCCAGCTCCAACTCCATCCAGCTCTACTACCGAAAGCTCTTCTGCTCCAGTATCCAGCTCCACCACTGAAAGCTCTTCTGCTCCAGCTCCAACTCCATCCAGCTCTACTACCGAAAGCTCTTCTGCTCCAGTAACCAGCTCCACCACTGAAAGCTCTTCTGCTCCAGTACCAACTCCATCAAGCTCTACTACTGAAAGCTCTTCTGCTCCAGTAACCAGCTCTACTACTGAAAGCTCTTCTGCTCCAGTACCAACTCCATCAAGCTCTACTACTGAAAGCTCTTCTGCTCCAGTAACCAGTTCCACCACTGAAAGCTCTTCTGCTCCAGTACCAACTCCATCCAGCTCTACCACTGAAAGCTCTTCTGCTCCAGCTCCAACTCCATCCAGCTCTACTACCGAAAGCTCTTCTGCTCCAGTATCCAGCTCCACCACTGAAAGCTCTTCTGCTCCAGCTCCAACTCCATCCAGCTCTACTACCGAAAGCTCTTCTGCTCCAGTAACCAGCTCCACCACTGAAAGCTCTTCTGCTCCAGTAACCAGCTCCACCACTGAAAGCTCTTCTGCTCCAGTACCAACTCCATCAAGCTCTACTACTGAAAGCTCTTCTGCTCCAGTAACCAGCTCCACCACTGAAAGCTCTTCTGCTCCAGTACCAACTCCATCAAGCTCTACTACTGAAAGCTCTTCTGCTCCAGTAACCAGCTCCACCACTGAAAGCTCTGTAGCACCAGTACCAACCCCATCTTCCTCTAGCAACATCACTTCCTCCGCTCCATCATCATCCAAATACCCTGGCAGTCAAACAGAAACCTCTGTTTCTTCTACAACCGAAACTACCATTGTTCCAACTACAACTACGACTTCTGTCACTACACCATCAACAACCACTATTACCACTACGGTTTGCTCTACAGGAACAAACTCTGCCGGTGAAACAACCTCTGGATGCTCTCCAAAGACCGTTACAACTACTGTTCCAACTACAACTACGACTTCTGTCACTACATCATCAACAACCACTATTACTACTACGGTTTGCTCTACAGGAACAAACTCTGCCGGTGAAACTACTTCTGGATGCTCTCCAAAGACCATTACAACTACTGTTCCATGTTCAACCAGTCCAAGCGAAACCGCCTCGGAATCAACAACCACTTCACCTACCACACCTGTAACTACAGTTGTCTCAACCACCGTCGTTACTACTGAGTATTCTACTAGTACAAAACCAGGTGGTGAAATTACAACTACATTTGTCACCAAAAACATTCCAACCACTTACCTAACCACAATTGCTCCAACTCCATCAGTCACTACGGTTACCAATTTCACCCCAACCACTATTACTACTACGGTTTGCTCTACAGGTACAAACTCTGCCGGTGAAACTACCTCTGGATGCTCTCCAAAGACTGTCACAACCACTGTTCCTTGTTCAACTGGTACTGGCGAATACACTACTGAAGCTACCACCCCTGTTACAACAGCTGTCACAACCACCGTTGTTACCACTGAATCATCTACGGGTACTAACTCCGCTGGTGAGACGACAACTGGTTACACAACAAAGTCTGTACCAACCACCTATGTAACCACTTTGGCTCCAAGTGCACCAGTAACTCCTGCCACTAATGCCGTACCAACTACAATAACCACTACTGAATGTTCTGCTGCTACAAACGCTGCCGGTGAAACTACATCTGTATGCTCTGCTAAGACTATCGTAAGTTCTGCAAGCGCAGGCGAAAACACCACCCCTGTCACGACAGCTGTCACAACCACCGTTGTTACCACTGAATCATCTACGGGTACTAACTCCGCTGGTGAGACGACAACTGGTTACACAACAAAGTCTGTACCAACCACCTATGTAACCACTTTGGCTCCAAGTGCACCAGTAACTCCTGCCACTAATGCCGTACCAACTACAATAACCACTACTGAATGTTCTGCTGCTACAAACGCTGCCGGTGAAACTACATCTGTATGCTCTGCTAAGACTATCGTAAGTTCTGCAAGCGCAGGCGAAAACACCACCCCTGTCACGACAGCTATTCCAACCACAGTTGTTACCACTGAGTCATCTGTTGGTACTAACTCCGCTGGCGAAACAACAACTGGTTACACAACCAAGTCCATCCCAACCACTTACATAACCACTTTGATTCCAGGTTCAAATGGTGCCAAGAATTACGAAACTGTGGCCACAGCAACCAACCCTATTTCAATCAAGACTACATCCCAACTAGCTACAACAGCTTCTGCTTCTAGCATGGCTCCCGTTGTCACATCTCCATCTCTAACTGGTCCACTACAATCTGCTTCTGGTTCTGCAGTCGCTACATACTCTGTTCCTTCTATCTCGAGTACTTACCAAGGTGCTGCTAATATCAAGGTTCTTGGAAACTTTATGTGGTTGCTACTCGCTCTTCCAGTTGTATTCTAA |

| **>*FLO11*_YPS128** |
| --- |
| ATGCAAAGACCATTTCTACTCGCTTATTTGGTCCTTTCGCTTCTATTTAACTCAGCTTTGGGTTTTCCAACTGCACTAGTTCCTAGAGGATCCTCCGAAGGAACTAGCTGTAATTCTATCGTTAATGGCTGTCCCAACTTAGACTTCAATTGGCACATGGACCAACAAAATATCATGCAGTATACTTTGGATGTGACTTCCGTTTCTTGGGTTCAAGACAACACATACCAAATCACTATTCATGTCAAAGGTAAAGAAAACATTGACCTAAAATATCTATGGTCTTTGAAAATCATTGGTGTCACTGGTCCAAAAGGTACCGTCCAACTATACGGTTACAACGAAAATACCTATTTGATTGACAACCCAACTGATTTCACAGCCACTTTTGAAGTCTATGCCACACAAGATGTCAACAGCTGTCAGGTGTGGATGCCTAACTTCCAAATTCAATTCGAGTATTTGCAAGGTAGTGCCGCTCAATATGCAAGCTCTTGGAAATGGGGAACTACATCTTTTGATTTGTCTACTGGTTGTAACAACTATGACAATCAAGGCCACTCTCAAACAGATTTCCCAGGCTTCTATTGGAACATAGATTGTGACAACAATTGTGGCGGTACGAAGTCATCTACCACTACATCAACTAGTACTTCCGAGTCATCTACCACTACATCAACTAGTACTTCCGAGTCATCTACCACTACATCAACTAGTACTTCCGAGTCATCTACCACTACATCAACTACCACTTCAGAGTCATCTACATCATCATCAACCACCGCTCCTGCTACACCAACCACTACCTCATGCACTAAGGAAAAGCCTACACCCCCAACCACTACCTCATGCACAAAGGAAAAGCCTACACCTCCTCATCACGACACCACTCCATGTACAAAGAAGAAAACCACCACATCTAAGACATGCACTAAGAAGACTACTACTCCAGTACCAACCCCATCAAGCTCTACTACTGAAAGCTCTTCTGCTCCAGTACCAACCCCATCAAGCTCTACCACTGAAAGCTCTTCTGCTCCAGTAACCAGCTCTACTACCGAAAGCTCTTCTGCTCCAGTACCAACTCCATCAAGCTCTACCACTGAAAGCTCTTCTGCTCCAGTACCAACTCCATCCAGCTCTACTACTGAAAGCTCTTCTGCTCCAGTACCAACTCCATCAAGCTCTACTACTGAAAGCTCCTCTGCTCCAGCTCCAACTCCATCCAGCTCCACTACTGAAAGCTCCTCTGCTCCAGTATCCAGCTCTACTACTGAAAGCTCTTCTGCTCCAGTACCAACTCCATCAAGCTCTACTACTGAAAGCTCCTCTGCTCCAGCTCCAACTCCATCCAGCTCCACTACTGAAAGCTCCTCTGCTCCAGTATCCAGCTCTACTACTGAAAGCTCTTCTGCTCCAGTACCAACTCCATCCAGCTCTACCACTGAAAGCTCTTCTGTTCCAGTACCAACCCCATCAAGCTCTACTACTGAAAGCTCTTCTGCTCCAGTACCAACCCCATCAAGCTCTACCACTGAAAGCTCTTCTGCTCCAGTAACCAGCTCTACTACCGAAAGCTCTTCTGCTCCAGCTCCAACTCCATCCAGCTCTACTACTGAAAGCTCTTCTGCTCCAGCTCCAACTCCATCCAGCTCTACTACTGAAAGCTCTTCTGCTCCAGTAACCAGCTCTACCACTGAAAGCTCTTCTGCTCCAGCTCCAACTCCATCCAGCTCCACCACTGAAAGCTCTTCTGCTCCAGTACCAACTCCATCAAGCTCTACCACTGAAAGCTCTTCTGCTCCAGTACCAACTCCATCAAGCTCCACTACTGAAAGCTCTTCTGCTCCAGCTCCAACTCCATCCAGCTCCACTACTGAAAGCTCCTCTGCTCCAGTATCCAGCTCTACTACTGAAAGCTCTTCTGCTCCAGTACCAACTCCATCCAGCTCTACCACTGAAAGCTCCTCTGCTCCAGTAACCAGCTCTACTACCGAAAGCTCTTCTGCTCCAGCTCCAACTCCATCAAGCTCTACTACTGAAAGCTCCTCTGCTCCAGTATCCAGCTCTACTACTGAAAGCTCTGTAGCACCAGTACCAACCCCATCTTCCTCTAGCAACATCACTTCCTCCGCTCCATCTTCAACTCCATTCAGCTCTAGCACTGAAAGCTCTTCTGTTCCAGTATCCAGCTCCACCACTGAAAGCTCTGTAGCACCAGTACCAACCCCATCTTCCTCTAGCAACATCACTTCCTCCGCTCCATCATCATCCAAATACCCTGGCAGTCAAACAGAAACCTCTGTTTCTTCTACAACCGAAACTACCATTGTTCCAACTACAACTACGACTTCTGTCACTACACCATCAACAACCACTATTACCACTACGGTTTGCTCTACAGGAACAAACTCTGCCGGTGAAACAACCTCTGGATGCTCTCCAAAGACCGTTACAACTACTGTTCCAACTACAACTACGACTTCTGTCACTACATCATCAACAACCACTATTACTACTACGGTTTGCTCTACAGGAACAAACTCTGCCGGTGAAACTACTTCTGGATGCTCTCCAAAGACCATTACAACTACTGTTCCATGTTCAACCAGTCCAAGCGAAACCGCCTCGGAATCAACAACCACTTCACCTACCACACCTGTAACTACAGTTGTCTCAACCACCGTCGTTACTACTGAGTATTCTACTAGTACAAAACCAGGTGGTGAAATTACAACTACATTTGTCACCAAAAACATTCCAACCACTTACCTAACCACAATTGCTCCAACTCCATCAGTCACTACGGTTACCAATTTCACCCCAACCACTATTACTACTACGGTTTGCTCTACAGGTACAAACTCTGCCGGTGAAACTACCTCTGGATGCTCTCCAAAGACTGTCACAACCACTGTTCCTTGTTCAACTGGTACTGGCGAATACACTACTGAAGCTACCACCCCTGTTACAACAGCTGTCACAACCACCGTTGTTACCACTGAATCCTCTACGGGTACTAACTCCGCTGGTGAGACGACAACTGGTTACACAACAAAGTCTGTACCAACCACCTATGTAACCACTTTGGCTCCAAGTGCACCAGTAACTCCTGCCACTAATGCCGTACCAACTACAATAACCACTACTGAATGTTCTGCTGCTACAAACGCTGCCGGTGAAACTACATCTGTATGCTCTGCTAAGACTATCGTAAGTTCTGCAAGCGCAGGCGAAAACACCACCCCTGTCACGACAGCTATTCCAACCACAGTTGTTACCACTGAGTCATCTGTTGGTACTAACTCCGCTGGCGAAACAACAACTGGTTACACAACCAAGTCCATCCCAACCACTTACATAACCACTTTGATTCCAGGTTCAAATGGTGCCAAGAATTACGAAACTGTGGCCACAGCAACCAACCCTATTTCAATCAAGACTACATCCCAACTAGCTACAACAGCTTCTGCTTCTAGCATGGCTCCCGTTGTCACATCTCCATCTCTAACTGGTCCACTACAATCTGCTTCTGGTTCTGCAGTCGCTACATACTCTGTTCCTTCTATCTCGAGTACTTACCAAGGTGCTGCTAATATCAAGGTTCTTGGAAACTTTATGTGGTTGCTACTCGCTCTTCCAGTTGTATTCTAA |

| **>*HPF1*_DBVPG6044** |
| --- |
| ATGGTCAAACCCATTGCTACACTTCAAGCCGTTTTGGCTTCGCTCCTTTACTCCCAAAGTAAATTGGGCCAATATTATACCCACAGTTCCTCAATCGCTAGTCACAGCTCCACTGCCGTTTCGTCAACTTCATCAGGTTCTGTTTCCATCAGTAGTTCTATTGTTGAGTCGACCTCATCTGCTTCTGATGTCTCGAGCTCTCTCACTGAGTTAACATCATCCTCCACCGAAGTCTCGAGCACCATTGCTCCATCAACCTCGTCCTCTGAAGTCTCGAGCTCTATTACTTCATCAGGCTCATCAGTCTCCGGCTCATCTTCTATTACTTCATCAGGCTCATCAGTCTCCAGTTCATCTTCTGTCACAGAATCAGGCTCATCCGCCCCAGGTTCATCTACTTCCATTACATCAGGTTCATCCTCCGCCACTGAATCAGGCTCATCAGTCTCCGGTTCATCTACTTCCATTACATCAGGCTCATCCTCCGCCACTGAATCAGGCTCATCAGTCTCTGGTTCATCTTCTGCCACAGAATCAGGCTCATCAGTCTCCGGTTCATCTACTTCCATTACATCAGGCTCATCCTCCGCCACTGAATCAGGCTCATCAGTCTCCGGTTCATCTACTTCCATTACATCAGGCTCATCCTCCGCCACTGAATCAGGCTCATCAGTCTCCGGTTCATCTACTTCCATTACATCAGGCTCATCCTCCGCCACTGAATCAGGCTCATCAGTCTCCGGTTCATCTACTTCCATTACATCAGGCTCATCCTCCGCCACTGAATCAGGCTCATCAGTCTCCGGTTCATCTACTTCCATTGCATCAGGCTCATCCTCCGCCACTGAATCAGGCTCATCAGTCTCCGGTTCATCTACTTCCATTACATTAGGCTCATCTTCTGTCACAGAATCAGGCTCATCAGTCTCCGGTTCATCTACTTCCATTACATCAGGCTCATCTTCTGTCACAGAATCAGGCTCATCCGCCCCAGGTTCATCTACTTCCATTACATCAGGCTCATCCTCTGCCACAGAATCAGGCTCATCCGCCCCAGGTTCATCTACTTCCATTACATCAGGTTCAACTTCTGTCATAGAATCAGGCTCATCAGTCTCCGGTTCATCTACTTCCATTACATCAGGCTCATCTTCTGTCACAGAATCAGGCTCATCCGCCCCAGGTTCATCTACTTCCATTACATCAGGCTCATCCTCTGCCACAGAATCAGGCTCATCCGCCCCAGGTTCATCTACTTCCATTACATCAGGCTCATCTTCTGTCACAGAATCAGGCTCATCCGCCCCAGGTTCATCTACTTCCATTACATCAGGCTCATCCTCTGCCACTGAATCAGGCTCATCCGCCCCAGGTTCATCTACTTCCATTACATCAGGTTCATCCTCCGCCACTGAATCAGGCTCATCCGCCCCAGGTTCATCTACTTCCATTACATCAGGTTCAACTTCTGCCACAGAATCAGGCTCATCCGCCCCAGGTTCATCTACTTCCATTACATCAGGTTCAACTTCTGCCACAGAATCAGGCTCATCCGCCTCCGGTTCATCCTCCGCCACAGAATCAGGCTCATCCGCCCCAGGTTCATCTACTTCCATTACATCAGGCTCATCTTCTGTCACAGAATCAGGCTCATCAGTCTCCGGTTCATCTACTTCCATTACATTAGGCTCATCTTCTGTCACAGAATCAGGCTCATCAGTCTCCGGTTCATCTACTTCCATTACATCAGGCTCATCTTCTGTCACAGAATCAGGCTCATCCGCCCCAGGTTCATCTACTTCCATTACATCAGGCTCATCCTCTGCCACAGAATCAGGCTCATCCGCCCCAGGTTCATCTACTTCCATTACATCAGGTTCAACTTCTGTCATAGAATCAGGCTCATCAGTCTCCGGTTCATCTACTTCCATTACATCAGGCTCATCTTCTGTCACAGAATCAGGCTCATCCGCCCCAGGTTCATCTACTTCCATTACATCAGGCTCATCCTCCGCCACAGAATCAGGCTCATCAGTCTCTGGTTCATCTTCTGCCACAGAATCAGGCTCATCAGTCTCCGGTTCATCTACTTCCATTACATCAGGCTCATCCTCCGCCACTGAATCAGGCTCATCAGTCTCCGGTTCATCTACTTCCATTACATCAGGCTCATCCTCCGCCACTGAATCAGGCTCATCAGTCTCCGGTTCATCTACTTCCATTACATCAGGCTCATCTTCTGCCACAGAATCAGGCTCATCAGTCTCCGGTTCATCTACTTCCATTACATCAGGTTCAACTTCTGCCACAGAATCAGGCTCATCCGCCTCCGGTTCATCTACTTCCATTACATTAGGCTCATCTTCTGTCACAGAATCAGGCTCATCAGTCTCCGGTTCATCTACTTCCATTACATCAGGCTCATCTTCTGTCACAGAATCAGGCTCATCAGTCTCCGGTTCATCTACTTCCATTACATCAGGCTCATCTTCTGTCACAGAATCAGGCTCATCAGTCTCCGGTTCATCTACTTCCATTACATTAGGCTCATCTTCTGTCACAGAATCAGGCTCATCAGTCTCCGGTTCATCTACTTCCATTACATCAGGCTCATCTTCTGTCACAGAATCAGGCTCATCCGCCCCAGGTTCATCTACTTCCATTACATCAGGCTCATCTTCTGTCACAGAATCAGGCTCATCCGCCCCAGGTTCATCTACTTCCATTACATCAGGCTCAACTTCTGCCACTGAATCAGGCTCATCCGCCCCAGGTTCATCTACTTCCATTACATCAGGTTCAACTTCTGCCACAGAATCAGGCTCATCCGCCTCCGGTTCATCTTCTGCCACAGAATCAGGCTCATCCGCCTCCGGTTCATCTTCTGCCACAGAATCAGGCTCATCCTCATCAGCATCTGAATCATCTATCACACAATCTGGTACCGCTTCCGGTTCATCAGCCTCCAGCACGTCCGGTTCTGTTACACAATCTGGTTCCTCCGTTTCCGGTTCATCAGCTTCTTCTGCTCCAGGTATCTCGAGTTCAATTCCTCAATCAACCTCATCGGCTTCCACTGCCTCCGGTTCTATCACCTCCGGTACCTTAAGTTCTATTACCTCTTCGGCTTCTAGTGCAACTGCAACTACTTCCAACTCTCTTTCTTCCAGCGACGGTACCGTTTACTTGCCATCCACAACAATTAGCGGTGATCTCAGAGTTACTGGTAAAGTAATTGCAACCGAGCCCGTGGAAGTCGCTGCCGGTGGTAAGTTGACTTTACTTGACGGTGAAAAATACGTCTTCTCATCTGATCTAAAAGTCTACGGTGACTTGCTTGTGAAAAAGTCCAAAGAAACCTATCCAGGTACCGAATTCGACATCTCCGGTGAAAACTTTGACGTGACCGGTAACTTCAACGCTGAAGAATCCGCTGCCACCTCTGCATCCATCTACTCCTTCACTCCAAGTTCTTTTGACAACAGTGGTGACATTTCCTTAAGTCTATCAAAGTCCAAGAAGGGTGAAGTCACTTTCTCTCCATACTCCAATTCTGGTGCCTTCTCTTTCTCGAACGCCATTCTCAACGGTGGTTCTGTTTCCGGTCTACAACGTAGAGACGACACTGAAGGTTCAGTAAACAACGGTGAAATTAACCTAGACAATGGAAGTACCTACGTCGTTGTCGAACCAGTTTCTGGAAGTGGTACAATCAACATCATCTCTGGCAACCTTTACTTGCACTATCCAGACACCTTTACTGGCCAAACTGTTGTATTCAAGGGTGAAGGTGTTCTTGCCGTTGACCCTACCGAAAGCAACACTACTCCTATCCCTGTGGTTGGATACACTGGTGAAAACCAAATCGCCATTACAGCAGATGTAACTGCTCTTTCTTACGACAGTGCTACTGGTGTTTTAACTGCAACACAAGGCAACTCACAATTCTCCTTCTCTATTGGTACTGGATTCTCCAGTTCTGGTTTCAACGTCTCCGAAGGAACATTTGCTGGTGCCTATGCTTATTATCTAAATTACGGAGGTGTTGTTGCTTCCAGCGCTACACCCTCATCCACATCTACCACATCAGGGGCTACCAACTCTACTTCCGGTTCCACTTCATTTGGTGCTTCCGTAACAGGTTCAACTGCCTCCACTTCATTCGGTGCTTCCGTAACTGGTTCAACCGCTTCCACTTCATTCGGTGCTTCCGTGACTGGTTCAACGGCTTCCACCTTGACTTCCGGCTCCCCATCTGTTTATACCACAACATTAACATATGCAACAACCACAAGCACAGTAGTTGTCTCCTGTTCAGAAACAACTGATTCGAACGGTAACGTCTATACCATTACCACAACCGTACCATGTTCATCTACCACCGCCACTATCACTTCTTGCGATGAGACCGGATGTCATGTAACTACGTCTACCGGTACCGTCGCCACTGAAACCGTTTCTTCCAAATCATACACCACTGTTACCGTCACCCACTGTGACAACAATGGCTGTAACACCAAGACTGTCACTTCTGAATGTCCTGAAGAAACTTCAGCAACTACTACTTCTCCAAAATCATACACTACTGTTACCGTTACTCACTGTGACGACAACGGCTGTAACACTAAGACTGTCACCTCTGAGGCCCCTGAAGCCACAACCACTACTGTTTCTCCAAAGACATACACTACCGCTACTGTTACTCAGTGCGATGACAATGGATGTAGCACCAAGACTGTCACTTCTGAAGCTCCTAAAGAAACTTCAGCAACTACTACTTCTCCAAAATCATACACTACTGTTACCGTTACTCACTGTGACGACAACGGCTGTAACACTAAGACTGTCACCTCTGAGGCCCCTGAAGCCACAACCACTACTGTTTCTCCAAAGACATACACTACCGCTACTGTTACTCAGTGCGATGACAATGGATGTAGCACCAAGACTGTCACTTCTGAAGCTCCTAAAGAAACTTCAGCAACTACTACTTCTCCAAAATCATACACTACTGTTACCGTTACTCACTGTGACGACAACGGCTGTAACACTAAGACTGTCACCTCTGAGGCCCCTGAAGCCACAACCACTACTGTTTCTCCAAAGACATACACTACCGCTACTGTTACTCAGTGCGATGACAATGGATGTAGCACCAAGACTGTCACTTCTGAAGCTCCTAAAGAAACTTCAGCAACTACTACTTCTCCAAAATCATACACTACTGTTACCGTTACTCACTGTGACGACAACGGCTGTAACACTAAGACTGTCACCTCTGAGGCCCCTGAAGCCACAACCACTACTGTTTCTCCAAAGACATACACTACCGCTACTGTTACTCAGTGCGATGACAATGGATGTAGCACCAAGACTGTCACTTCTGAAGCTCCTAAAGAAACTTCAGCAACTACTACTTCTCCAAAATCATACACTACTGTTACCGTTACTCACTGTGACGACAACGGCTGTAACACTAAGACTGTCACCTCTGAGGCCCCTGAAGCCACAACCACTACTGTTTCTCCAAAGACATACACTACCGCTACTGTTACTCAGTGCGATGACAATGGATGTAGCACCAAGACTGTCACTTCTGAAGCTCCTAAAGAAACTTCAGCAACTACTACTTCTCCAAAATCATACACTACTGTTACCGTTACTCACTGTGACGACAACGGCTGTAACACTAAGACTGTCACCTCTGAGGCCCCTGAAGCCACAACCACTACTGTTTCTCCAAAGACATACACTACCGCTACTGTTACTCAGTGCGATGACAATGGATGTAGCACCAAGACTGTCACTTCTGAAGCTCCTAAAGCAACCTCATTGACTACTGCCATTTCCAAGGCTTCTAGTGCAATTTCCACATACTCCAAATCTGCAGCTCCAATAAAGACCTCTACTGGTATCATTGTCCAGTCCGAGGGTATTGCCGCAGGATTGAATGCCAATACTTTGAATGCATTGGTCGGTATTTTCGTTCTTGCTTTCTTTAACTAA |

| **>*HPF1*_YPS128** |
| --- |
| ATGTTCAATCGCTTTAATAAACTTCAAGCCGCTTTGGCTTTGGTCCTTTACTCCCAAAGTGCATTGGGCCAATATTATACCAACAGTTCCTCAATCGCTAGTAACAGCTCCACTGCCGTTTCGTCAACTTCATCAGGTTCTGTTTCCATCAGTAGTTCTATTGTTGAGTCGACCTCATCTGCTTCTGATGTCTCGAGCTCTCTCACTGAGTTAACATCATCCTCCACCGAAGTCTCGAGCACCATTGCTCCATCAACCTCGTCCTCTGAAGTCTCGAGCTCTATTACTTCATCAGGCTCATCAGTCTCCGGCTCATCTTCTATTACTTCATCAGGCTCATCAGTCTCCAGTTCATCTTCTGTCACAGAATCAGGCTCATCCGCCCCAGGTTCATCTACTTCCATTACATCAGGTTCATCTTCTGCCACAGAATCAGGCTCATCAGTCTCCGGTTCATCTACTTCCATTACATCAGGCTCATCCTCCGCCACTGAATCGGGCTCATCAGTCTCCGGTTCAACTTCTGCCACTGAATCAGGCTCATCCGCCTCCGGTTCAACTTCCGCCACTGAATCAGGCTCATCAGTCTCCGGTTCATCTTCTGCCACAGAATCAGGCTCATCAGTCTCCGGTTCATCTACTTCCATTACATCAGGCTCATCCTCCGCCACTGAATCAGGCTCATCCGCCTCCGGTTCAACTTCCGCCACAGAATCAGGCTCATCCGCCTCCGGTTCATCTTCTGCCACTGAATCAGGCTCCGCTTCTTCGGTTCCTAGCTCATCCGGTTCTATCACAGAATCAGGCTCATCCTCATCAGCATCTGAATCATCTATCACACAATCTGGTACCGCTTCCGGTTCATCAGCCTCCAGCACGTCCGGTTCTGTTACACAATCTGGTTCCTCCGTTTCCGGTTCATCAGCTTCTTCTGCTCCAGGTATCTCGAGTTCAATTCCTCAATCAACCTCATCGGCTTCCACTGCCTCCGGTTCTATCACCTCCGGTACCTTAAGTTCTATTACCTCTTCGGCTTCTAGTGCAACTGCAACTGCTTCCAACTCTCTTTCTTCCAGCGATGGTACTATTTATTTGCCTTCTACAACCATCAGTGCTGACATCACACTCACCGGTTCAGTCATTGCAACTGAAGCTGTCGAAGTCGCTGCAGGTGGTAAGTTGACCCTACTTGATGGTGACAAATACGTTTTTTCTGCTGATTTCATAATCCATGGTGGCGTTTTCGTAGAAAAGTCTAAGCCAACTTACCCAGGTACCGAATTCGACATTTCTGGTGAAAACTTTGATGTATCTGGTACCTTTAACGCTGAAGAGCCTGCTGCTTCTTCCGCATCTGCATACTCCTTCACTCCAGGCTCTTTCGATAACAGTGGTGATATTTCTTTGAGTCTATCAGAGTCCACAAAGGGCCAAGTCACATTCTCTCCTTACTCTAACTCTGGTGCTTTCTCTTTCTCAAATGCTATTCTCAATGGTGGTTCCGTCTCTGGTTTGCAACGTAGAGCTGAATCAGGTTCTGTCAACAACGGTGAGATAAATATTGAGAATGGCAGTACCTACGTCGTTGTCGAACCAGTTTCTGGAAGTGGTACAATCAACATCATCTCTGGCAACCTTTACTTGCACTATCCAGACACCTTTACTGGCCAAACTGTTGTATTCAAGGGTGAAGGTGTTCTTGCCGTTGACCCTACCGAAAGCAACACTACCCCTATCCCTGTGGTTGGATACACTGGTGAAAACCAAATCGCCATTACAGCAGATGTAACTGCTCTTTCTTACGACAGTGCTACTGGTGTTTTAACTGCAACACAAGGCAACTCACAATTCTCCTTCTCTATTGGTACTGGATTCTCCAGTTCTGGTTTCAACGTCTCCGAAGGAACATTTGCTGGTGCCTATGCTTATTATCTAAATTACGGAGGTGTTGTTGCTTCCAGCGCTACACCCTCATCCACATCTACCACATCAGGGGCTACCAACTCTACTTCCGGTTCCACTTCATTTGGTGCTTCCGTAACAGGTTCAACTGCCTCCACTTCATTCGGTGCTTCCGTAACTGGTTCAACCGCTTCCACTTCATTCGGTGCTTCCGTGACTGGTTCAACGGCTTCCACCTTGACTTCCGGCTCCCCATCTGTTTATACCACAACATTAACATATGCAACAACCACAAGCACAGTAGTTGTCTCCTGTTCAGAAACAACTGATTCGAACGGTAACGTCTATACCATTACCACAACCGTACCATGTTCATCTACCACCGCCACTATCACTTCTTGCGATGAGACCGGATGTCATGTAACTACGTCTACCGGTACCGTCGCCACTGAAACCGTTTCTTCCAAATCATACACCACTGTTACCGTCACCCACTGTGACAACAATGGCTGTAACACCAAGACTGTCACTTCTGAATGTCCTGAAGAAACTTCAGCAACTACTACTTCTCCAAAATCATACACTACTGTTACCGTTACTCACTGTGACGACAACGGCTGTAACACTAAGACTGTCACCTCTGAGGCCCCTGAAGCCACAACCACTACTGTTTCTCCAAAGACATACACTACCGCTACTGTTACTCAGTGCGATGACAATGGATGTAGCACCAAGACTGTCACTTCTGAAGCTCCTAAAGAAACTTCAGAAACTTCAGAAACCAGTGCTGCCCCTAAGACATACACTACTGCCACTGTTACTCAATGTGATGACAATGGTTGTAACGTCAAGATAATCACCTCTCAAATACCTGAAGCTACTTCAACCGTCACCGCAACTAGTGCTTCTCCAAAGTCATACACTACTGTCACTTCTGAGGGTTCTAAAGCAACCTCATTGACTACTGCCATTTCCAAGGCTTCTAGTGCAATTTCCACATACTCCAAATCTGCAGCTCCAATAAAGACCTCTACTGGTATCATTGTCCAGTCCGAGGGTATTGCCGCAGGTTTGAATGCCAATACTTTGAATGCATTGGTCGGTATTTTCGTTCTTGCTTTCTTTAACTAA |
